# Discovery of an SQS-PSY Domain-Containing Protein in *Meloidogyne incognita* Reveals Its Function in Parasitism

**DOI:** 10.3390/ijms26189113

**Published:** 2025-09-18

**Authors:** Junru Lu, Runmao Lin, Yunlong Ma, Xin Sun, Yang Jiao, Xinyue Cheng, Bingyan Xie

**Affiliations:** 1 College of Life Sciences, Beijing Normal University, Beijing 100875, China; ximizhen@foxmail.com (J.L.); sunxin_lois@outlook.com (X.S.); 2 Institute of Vegetables and Flowers, Chinese Academy of Agricultural Sciences, Beijing 100081, China; jiaoyang_0425@163.com; 3 Key Laboratory of Green Prevention and Control of Tropical Plant Diseases and Pests, Ministry of Education, School of Tropical Agriculture and Forestry, Sanya Institute of Breeding and Multiplication, Hainan University, Sanya 572025, China; linrm2010@163.com; 4 Center for Biosafety, Chinese Academy of Inspection and Quarantine, Sanya 572024, China; mylsfh@126.com

**Keywords:** root-knot nematode, gene expression pattern, in situ hybridization, heterologous expression, host-induced gene silencing (HIGS), experimentally functional analysis, parasitism, differentially expressed genes (DEGs), nematode-plant interaction

## Abstract

Proteins containing the SQS-PSY domain, which include squalene synthetase (SQS), phytoene synthetase (PSY), and NDUFAF6, are functionally important and widely distributed in plants and animals. However, they have not been previously reported in nematodes. In this study, we identified a gene (*Minc31999*) encoding an SQS-PSY domain-containing protein in the root-knot nematode *Meloidogyne incognita*. In silico comparison and enzymatic assays of the recombinant protein indicated that this nematode protein is a putative NDUFAF6 homolog. Phylogenetic analysis revealed that this protein is evolutionarily conserved within the Nematoda phylum. RT-qPCR analysis showed that *Minc31999* is highly expressed during the early infection stage of *M. incognita*. Targeting the nematode gene *Minc31999* via host-induced gene silencing (HIGS) significantly hindered nematode development and virulence. In contrast, heterologous expression of *Minc31999* in *Arabidopsis thaliana* disrupted normal plant development and increased host susceptibility to nematode infection. Transcriptomic profiling (RNA-seq) of these transgenic plants prior to infections showed a widespread differential expression of genes across multiple metabolic pathways. We propose that this nematode SQS-PSY domain-containing protein may function as an effector that rewires host secondary metabolism to establish a parasitic relationship. Our study elucidates a novel strategy in nematode–plant interactions and advances our understanding of the functional evolution of SQS-PSY domain-containing proteins.

## 1. Introduction

The southern root-knot nematode (RKN), *Meloidogyne incognita*, is one of the most devastating plant parasitic nematodes and is widely endemic in subtropical and tropical regions. This obligatory sedentary parasite can infect almost all cultivated plants and is responsible for significant global agricultural losses. *M. incognita* is characterized by mitotic parthenogenesis and allopolyploidy [1]. Most populations are (hypo)triploid, while a few are diploid [2]. Parthenogenesis and polyploidy may provide the nematode with a stronger survival advantage and a high capacity to adapt to environmental selection. Understanding its pathogenic mechanisms is crucial for developing new control technologies. Since the publication of the first *M. incognita* genome [3], considerable effort has been devoted to exploring its pathogenicity. Numerous effectors have been identified from genomic, transcriptomic, and proteomic data by bioinformatic and experimental approaches [4,5,6,7]. Currently, seven *M. incognita* genomes have been deposited in NCBI. However, the molecular pathogenic mechanisms of *M. incognita* are not yet fully understood.

During the genomic analysis of *M. incognita*, we identified a gene (*Minc31999*) encoding a protein that contains a conserved SQS-PSY domain (squalene synthase/phytoene synthase, PF00494 in Pfam annotation). This gene is also predicted by antiSMASH (https://antismash.secondarymetabolites.org, accessed on 25 August 2020) to be involved in secondary metabolite biosynthesis, specifically as a biosynthetic terpene/phytoene synthase. Squalene synthetase (SQS) and phytoene synthetase (PSY) are bifunctional enzymes that catalyze the formation of a cyclopropane intermediate and its rearrangement to produce a straight-chain hydrophobic product, either squalene or phytoene [8]. Squalene synthetase condenses two farnesyl diphosphate (FPP, C15) molecules in a head-to-head (1′−1) manner to form a stable cyclopropane intermediate, pre-squalene diphosphate. This intermediate is then reduced using a pyridine dinucleotide cofactor to generate the final product, squalene (C30) [8]. This reaction constitutes the first committed step in the cholesterol biosynthetic pathway. Cholesterol, a precursor of steroid hormones and bile acids, plays essential roles in animal development and disease progression [9]. However, nematodes are known to be incapable of de novo sterol biosynthesis because they lack several enzymes in the canonical cholesterol biosynthesis pathway [10]. Consequently, their cholesterol supply depends entirely on exogenous absorption. Similarly, phytoene synthase catalyzes the head-to-head (1′−1) condensations of two geranylgeranyl diphosphate (GGPP, C20) molecules to produce phytoene (C40) [8]. This enzyme is crucial for carotenoid biosynthesis, which participates in photosynthesis, provides photoprotection, and supplies the precursor for strigolactone and abscisic acid (ABA) biosynthesis [11]. Although phytoene synthase is well-studied in plants, it is seldom reported in animals. Recently, a human gene (*NDUFAF6/c8orf38*) was found to encode a protein (NDUFAF6, NADH:ubiquinone oxidoreductase complex assembly factor 6) that also belongs to the PF00494 family, containing an SQS-PSY domain [12]. NDUFAF6 localizes to the mitochondria and contains a predicted phytoene synthase domain. It plays a critical role in assembling complex I of the mitochondrial respiratory chain by regulating the biogenesis of the ND1 subunit. Mutations in the NDUFAF6 gene can lead to complex I deficiency, which is associated with a range of human diseases [12,13,14]. A homolog of human NDUFAF6 was also identified in the fruit fly (*Drosophila melanogaster*), and it was suggested that most metazoans (but not all) possess an NDUFAF6 orthologue, but complex I biogenesis in some organisms does not require this protein [13]. However, no squalene synthetase, phytoene synthetase, or NDUFAF6 orthologs have been reported in nematodes to date.

In this study, we employ an integrated computational and experimental approach to characterize the SQS-PSY domain-containing protein encoded by *Minc31999* in the RKN *M. incognita*. First, we perform a comparative in silico analysis with known SQS-PSY domain-containing proteins and assess its enzymatic activity in vitro to determine whether it functions as a squalene or phytoene synthase. We then use RT-qPCR and in situ RNA hybridization to profile its expression in nematode life stages and tissues. To elucidate its biological functions, we conduct RNA interference (RNAi) and overexpression assays. Finally, we perform RNA-seq analysis on transgenic plant lines to identify host gene expression changes triggered by this nematode protein. Our goal is to classify this protein within the SQS-PSY family and elucidate its functional role in the nematode parasitism.

## 2. Results

### 2.1. Feature of SQS-PSY Domain-Containing Protein Encoded by Minc31999

**Figure 1:**
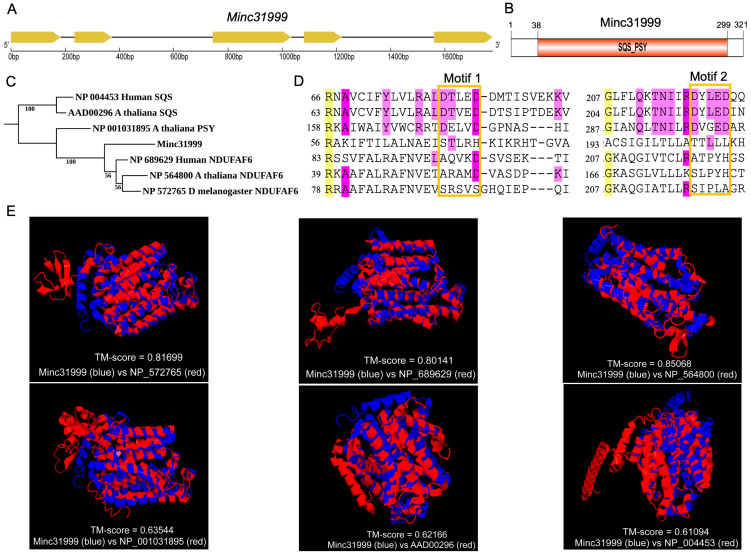
Structural and evolutionary analysis of the protein encoded by *Minc31999* in *M. incognita.* (**A**) Genomic organization showing five exons (boxes) and four introns (lines). (**B**) Domain architecture of the encoded protein with the conserved SQS-PSY domain. (**C**) Maximum likelihood phylogenetic tree constructed with IQ-TREE (1000 bootstraps) demonstrating evolutionary relationships among SQS-PSY domain-containing proteins from four species. (**D**) MUSCLE alignment of catalytic regions, highlighting the conserved active sites with yellow (identity ≥ 90%), dark purple (≥80%), and light purple (≥70%). The two characteristic DxxxD motifs are in orange boxes. (**E**) Predicted tertiary structure comparison between the protein encoded by *Minc31999* and known SQS-PSY domain-containing proteins by TM-align. The TM score value was used to assess the structural alignment between two proteins. It ranges from 0 to 1, where 1 means the structures are identical. NP_572765: fruit fly NDUFAF6; NP_689629: human NDUFAF6; NP_564800: *A. thaliana* NDUFAF6; NP_001031895: *A. thaliana* PSY; AAD00296: *A. thaliana* SQS; NP_004453: human SQS.

The full-length sequence of the *Minc31999* gene is 1790 bp, comprising five exons and four introns (Figure 1A). Its coding sequence (CDS) is 963 bp long and encodes a protein of 320 amino acid residues (Supplementary Data). A BLASTp analysis revealed that its best hit is an unnamed protein from *Meloidogyne enterolobii* (CAD2175695), another important RKN, with 96.56% amino acid identity (E-value < 1 × 10^−300^). Pfam annotation indicates that the protein belongs to the squalene/phytoene synthase protein family (PF00494). Furthermore, an NCBI conserved domain (CD) search predicted that the protein contains a conserved domain from the Isoprenoid_Biosyn_C1 superfamily (cl00210), which includes isoprenoid biosynthesis enzymes class 1 and the superfamily of trans-isoprenyl diphosphate synthases (trans_IPPS) (Figure 1B).

Then, we compared the nematode protein with those of known SQS-PSY domain-containing proteins from the three model species (*Homo sapiens*, *Drosophila melanogaster* and *Arabidopsis thaliana*). These included two squalene synthases (NP_004453, AAD00296), one phytoene synthase (NP_001031895), and three NDUFAF6 proteins (NP_689629, NP_572765, NP_564800). Our analysis revealed that the sequence identity between the nematode protein and each of the aforementioned proteins is less than 25%. A phylogenetic tree constructed from these sequences showed that the three NDUFAF6 proteins and the two squalene synthases each formed distinct, independent clades. The nematode protein and the phytoene synthase were located between these two clades, with the nematode protein being phylogenetically closer to the NDUFAF6 clade than to the SQS clade (Figure 1C). Given that *trans*_IPPS_HH proteins typically contain two characteristic aspartate-rich motifs (DxxxD) that mediate substrate binding and catalysis using bridging Mg^2+^ ions [13], we compared the catalytic sites following multiple sequence alignments. The results show that the two typical aspartate-rich active sites (DxxxD) are present in SQS and PSY sequences but are absent in the NDUFAF6 protein. In human and *Arabidopsis* NDUFAF6, the two DxxxD motifs are replaced with AxxxD (first motif) and AxxxH/SxxxH (second motif). However, these specific replacements were not found in the *Drosophila* NDUFAF6 or the *Minc31999*-encoded nematode protein. Instead, the matched motifs are SRSVS (first) and SIPLA (second) in *Drosophila* NDUFAF6 and STLRH (first) and TTLLL (second) in the nematode protein (Figure 1D). Using TM-align, we compared the predicted spatial structures of the SQS-PSY domain-containing proteins. The *Minc31999*-encoded nematode protein exhibited high structural similarity to NDUFAF6 proteins (TM score > 0.8) but low similarity to SQS or PSY (TM score < 0.64) (Figure 1E, Supplementary Figure S1), indicating a closer phylogenetic relationship to NDUFAF6.

### 2.2. Heterologous Expression of the SQS-PSY Domain-Containing Protein Encoded by Minc31999 and Catalytic Activity Assay

**Figure 2:**
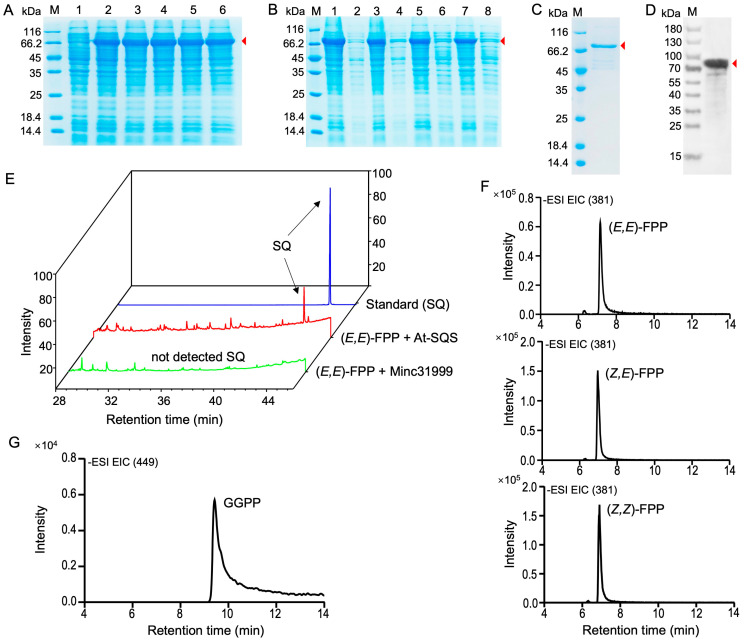
Heterologous expression of *Minc31999* gene in *E. coli* and enzymatic assay of recombinant protein. (**A**) Coomassie-stained SDS-PAGE showing recombinant protein expression in *E. coli*. Lane M: protein marker; Lane 1: uninduced sample; Lanes 2–6: induced samples. Red arrow indicates 81 kDa target protein. (**B**) Solubility analysis under different induction conditions (16 °C/37 °C; 0.2/1 mM IPTG). Odd lanes: pellet fractions; even lanes: soluble fractions. Lanes 1–4: induction at 37 °C; Lanes 5–8: induction at 16 °C; Lanes 1, 2, 5, 6: induction with 1 mM IPTG; Lanes 3, 4, 7, 8: induction with 0.2 mM IPTG. (**C**,**D**) Validation of purified recombinant protein by SDS-PAGE (**C**) and Western blot (**D**). (**E**) GC-MS analysis of squalene (SQ) production: blue (standard), red (*A. thaliana* SQS positive control), green (Minc31999 protein reaction). Substrate: (*E,E*)-FPP. (**F**,**G**) LC-MS detection of substrate consumption with FPP isomers (**F**) or GGPP (**G**). No new compound was detected except for substrate FPP or GGPP.

To determine whether the nematode protein encoded by *Minc31999* possesses squalene synthase or phytoene synthase catalytic activity, the full-length CDS was cloned and inserted into the pMAL-c5X vector. The recombinant vector was then transformed into *E. coli* for heterologous expression. After induction with 0.5 mM IPTG at 37 °C for 3 h, the target protein was clearly detected by SDS-PAGE and Coomassie blue staining (Figure 2A). We further assessed protein expression using different IPTG concentrations (0.2 mM and 1 mM) and temperatures (16 °C and 37 °C). SDS-PAGE analysis revealed no significant difference in the amounts of the recombinant protein produced under these various conditions. Although the recombinant protein was primarily found in inclusion bodies within the pellet fraction, a soluble form was also present in the supernatant (Figure 2B). This soluble recombinant protein was subsequently purified and confirmed by SDS-PAGE (Figure 2C) and Western blot analysis using an antiMBP monoclonal antibody (Figure 2D). Concurrently, the CDS of the *A. thaliana AT4G34640* gene, which encodes a squalene synthase (AAD00296), was cloned into the pET-30a vector and expressed in *E. coli* (Supplementary Figure S2A). This recombinant protein was purified using His-tag affinity column chromatography (Supplementary Figure S2B) and was used as a positive control for the SQS activity assay.

We then performed an enzyme activity assay for the recombinant protein using FPP and GGPP as substrates. First, we incubated (*E,E*)-FPP as a substrate with the purified recombinant proteins from *Minc31999* and *AT4G34640* (in a reaction solution containing Mg^2+^ ions) at 30 °C for 2 h. The products were then extracted and analyzed by gas chromatography–mass spectrometry (GC-MS) and liquid chromatography–mass spectrometry (LC-MS). The results show that squalene was detected in the reaction containing the *A. thaliana* SQS protein but not in the reaction with the nematode protein (Figure 2E). We also tested the enzymatic activity of the nematode protein using the other two FPP isomers ((*Z,Z*)-FPP and (*Z,E*)-FPP) and GGPP as substrates. Similarly, LC-MS analysis detected no new compound; only the substrates themselves were observed (FPP: a peak at *m*/*z* 381; GGPP: a peak at *m*/*z* 449) (Figure 2F,G). These results indicate that the nematode protein encoded by *Minc31999* lacks catalytic activity as either a squalene synthase or a phytoene synthase. Therefore, it is evolutionarily and functionally distinct from the canonical *trans*-IPPS HH family of proteins.

### 2.3. Discovery and Evolutionary Analysis of SQS-PSY Domain-Containing Proteins in Nematoda

**Figure 3:**
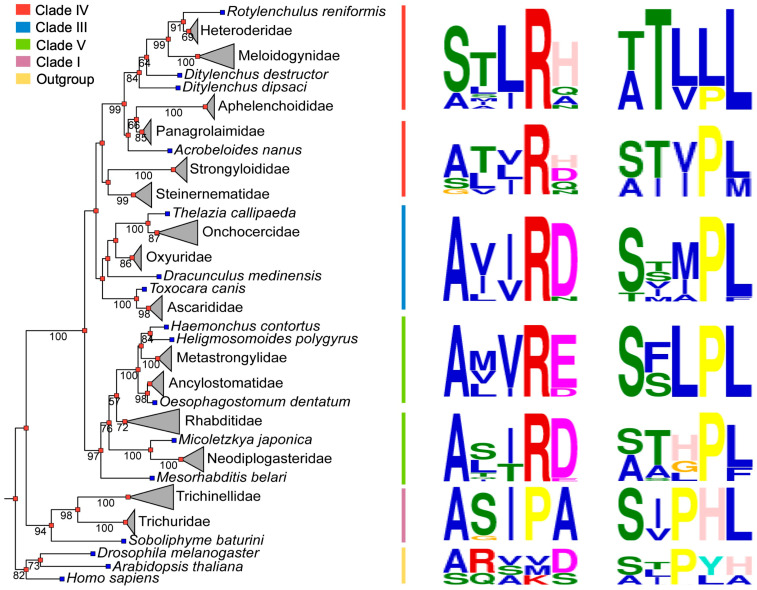
Phylogenetic relationship and motif conservation of nematode SQS-PSY domain-containing proteins. Maximum likelihood tree was constructed form the SQS-PSY domain sequences using IQ-TREE, with three non-nematode NDUFAF6 proteins as out-groups. Branch labels indicate bootstrap support >50%. Colored bars indicate major nematode clades. Right panel: Sequence logos illustrating the two characteristic motifs of the SQS-PSY domain, generated by MEME. The height of each amino acid symbol represents its relative frequency at that position.

To investigate the distribution and evolutionary relationship of SQS-PSY domain-containing proteins within the phylum Nematoda, we searched for homologous sequences of the protein encoded by *Minc31999* using BLASTp against all available nematode genomes in NCBI and WormBase. We retrieved a total of 96 SQS-PSY domain-containing protein sequences from various nematodes, which belong to clades I, III, IV, and V in current phylogenetic classifications (Supplementary Table S1), with one member identified per genome. A phylogenetic tree was constructed using the SQS-PSY domain sequences from these nematode proteins, along with NDUFAF6 sequences from humans, fruit flies, and *Arabidopsis*. Sequences with incomplete SQS-PSY domains were excluded from the analysis. The results show that the three non-nematode NDUFAF6 proteins form a distinct clade. Using this non-nematode NDUFAF6 clade as an outgroup, the phylogenetic relationship of the nematode proteins was found to be largely consistent with the established nematode evolutionary relationship based on small subunit ribosomal RNA (SSU rDNA) gene sequences [15,16]. Specifically, nematodes from clades I, III, IV, and V each form independent, monophyletic groups (Figure 3, Supplementary Figure S3). We also compared the two characteristic motifs within the SQS-PSY domain. The motifs were highly conserved within each genus; for example, *Meloidogne* spp. feature STLRH and TTLLL as the first and second motifs, respectively, while *Caenorhabditis* spp. feature ASIRD and THPLL. Furthermore, the predominant first and second motifs for each nematode clade were identified as follows: clade I: AxxxA and SxxxL; clade III: AxxxD and T/V/I/S/MxxxL; clade IV: SxxxH and TxxxL; and clade V: AxxxD/E and T/A/S/FxxxL (Figure 3). The results indicate that SQS-PSY domain-containing proteins are universal and evolutionarily conserved within the phylum Nematoda.

### 2.4. Expression Patterns of Minc31999 in M. incognita

To analyze the expression of *Minc31999* during *M. incognita* development, we analyzed publicly available transcriptomes from five life stages (egg, J2, J3, J4, and female adult, from the NCBI database, Table 1). BLASTn analysis using *Minc31999* CDS revealed homologous transcripts in all stages (Table 1), demonstrating that this gene is expressed throughout the nematode’s life cycle of *M. incognita*.

**Figure 4:**
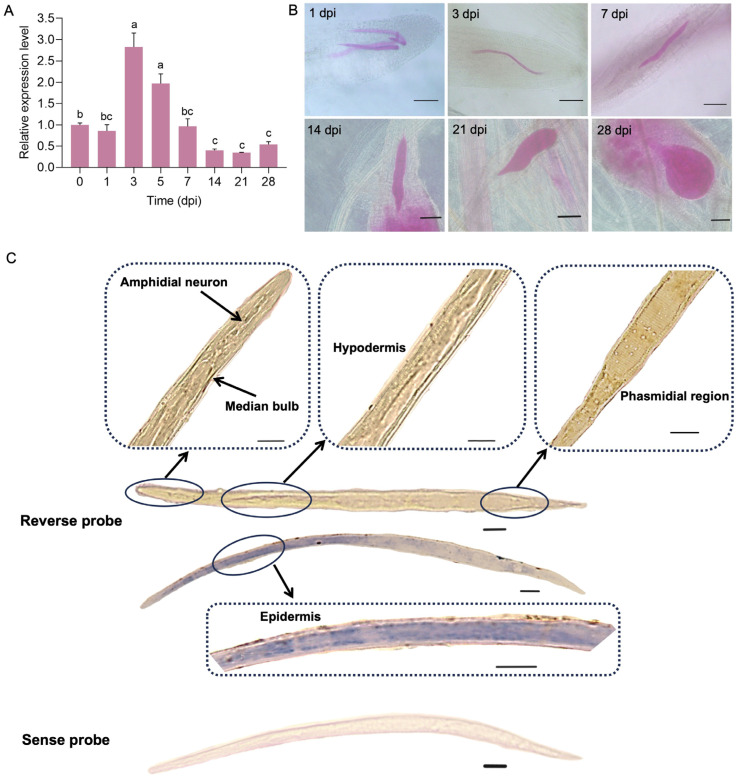
Expression analysis of *Minc31999* in *M. incognita*. (**A**) Quantitative expression analysis during the nematode infection on *A. thaliana* (0–28 dpi). Three biological replicates were analyzed by RT-qPCR with GAPDH coding gene as an internal control. Statistical significance (*p* < 0.05) was determined by one-way ANOVA with Games–Howell’s test. Different superscripts indicate significant differences. Error bars show standard errors (SEs). (**B**) Representative images of nematode developmental stages corresponding to expression time points. Root samples were stained with 0.1% acid fuchsin (nematodes appear pink). Scale bar = 100 μm. (**C**) Tissue-specific RNA localization detected by DIG in situ hybridization. Hybridization signals (brown) in amphidial neuron, phasmidial region, epidermis, and hypodermis are magnified. No hybridization signal is observed in sense probe control. Scale bar = 10 μm.

Then, after the inoculation of freshly hatched *M. incognita* J2 juveniles (J2s) onto the roots of *A. thaliana* (WT), the expression levels of *Minc31999* in the nematode were detected by RT-qPCR at 1, 3, 5, 7, 14, 21, and 28 days post-inoculation (dpi). Pre-parasitic J2 juveniles (pre-J2s, 0 dpi) were used as the control. The results show that the gene expression level was significantly upregulated after inoculation for 3 to 5 days, peaking at 3 dpi. Expression then decreased to the control level (pre-J2s) by 7 dpi and fell below the control level at 14 dpi and 28 dpi (Figure 4A). We also monitored nematode development at these time points and found that the nematodes were in the J2 stage from 1 to 7 dpi, subsequently developing into J3, J4, and adult stages at 14, 21, and 28 dpi, respectively (Figure 4B). The high expression level of *Minc31999* in the early parasitic J2 stage suggests that this gene may be involved in nematode parasitism. Furthermore, we determined the expression site of *Minc31999* in *M. incognita* using in situ hybridization (ISH). The results show that hybridization signals were detected throughout the entire nematode body, including in amphidial and phasmidial regions, the epidermis, and the hypodermis (Figure 4C).

### 2.5. RNAi Minc31999 by Host-Induced Gene Silencing Impacts M. incognita Development and Parasitism

**Figure 5:**
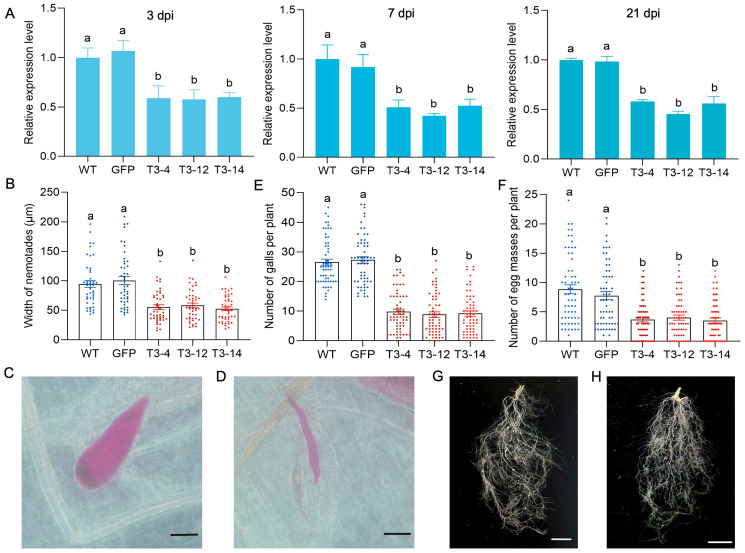
Host-delivered RNAi of *Minc31999* impairs *M. incognita* parasitism. (**A**) Relative expression of *Minc31999* in *M. incognita* parasitizing on transgenic *Arabidopsis* lines (T3-4/12/14 represent three independent transgenic lines, T3 generation) versus controls (WT and GFP line) at three infection stages (3/7/21 dpi). (**B**) Body width of *M. incognita* at 28 dpi (*n* = 45 individuals). (**C**,**D**) Representative images of nematode morphology, showing normal development parasitizing on WT (**C**) vs. delayed growth parasitizing on transgenic *A. thaliana* (**D**) at 28 dpi. Scale bar = 100 μm. (**E**,**F**) Quantification of root galls (**E**) and egg masses (**F**) at 42 dpi (*n* = 60 plants). (**G**,**H**) Macroscopic root damage comparison, WT (**G**) vs. transgenic line (**H**). Scale bar = 1 cm. Error bars represent SE. Different superscripts indicate significant differences (Tukey’s test for A/E; Games–Howell’s test for B/F; *p* < 0.05).

Host-induced gene silencing (HIGS) was employed to assess the biological function of *Minc31999* in the nematode *M. incognita*. A pSuper-*Minc31999*-RNAi vector was constructed and then transformed into *A. thaliana* by *Agrobacterium tumefaciens*-mediated transformation (ATMT) using the floral dip method, followed by selection on hygromycin. Positive transgenic *A. thaliana* lines were verified by PCR (Supplementary Figure S4). Subsequently, freshly hatched J2 juveniles of *M. incognita* were inoculated onto the roots of T3 transgenic lines, with GFP transgenic and wild-type (WT) *A. thaliana* plants serving as controls. The expression levels of *Minc31999* in the nematodes were detected by RT-qPCR at 3, 7, and 21 dpi. The results show that *Minc31999* expression in nematodes feeding on the RNAi transgenic lines was significantly reduced due to host-derived silencing compared to nematodes feeding on the control plants (Figure 5A). We also examined nematode development in different *A. thaliana* lines at 28 dpi. The results show that the body size (width) of female nematodes developing on the RNAi transgenic lines (mean width 52.6 to 58.3 μm) was significantly smaller than that of nematodes on WT (mean 94.5 μm) or GFP control plants (mean 100.5 μm) (Figure 5B–D).

We then observed the effects of HIGS on nematode parasitism. After inoculating *M. incognita* J2s onto roots of transgenic and control plants for 42 days, we examined the phenotypes of the different *A. thaliana* lines. This included quantifying the number of galls and egg masses, as well as assessing plant growth. The results show that the number of galls on the roots of transgenic lines (mean of nine galls/plant) was significantly reduced compared to those on control plants (mean of 27 galls/plant) (Figure 5E). Similarly, the number of egg masses on transgenic lines (mean of four masses /plant) was significantly lower than on the control groups (mean of nine masses/plant for WT and eight masses/plant for GFP) (Figure 5F). Consequently, root damage from *M. incognita* was visibly less severe in the transgenic lines than in the controls (Figure 5G,H). In contrast, the infection had no significant impact on the growth of aboveground plant parts or on root weight between the transgenic and control plants (Supplementary Figure S5).

### 2.6. Overexpression of Minc31999 Gene in A. thaliana May Impact Plant Growth and Resistance Against Nematode M. incognita

**Figure 6:**
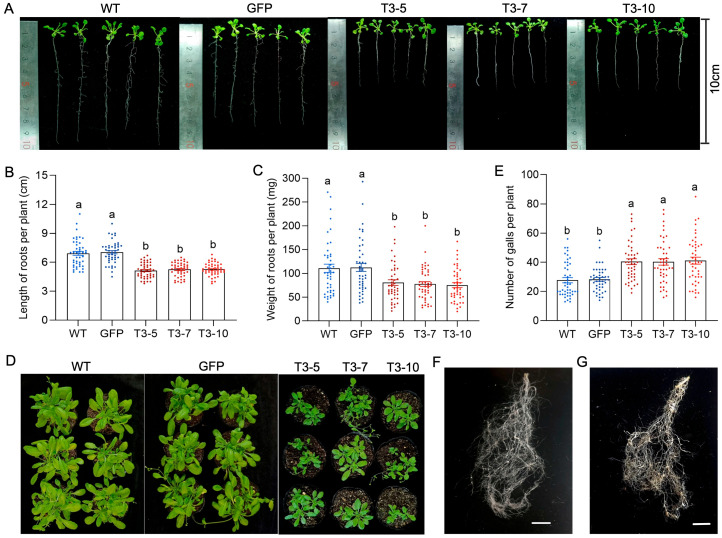
Overexpression of *Minc31999* in *A. thaliana* reduces plant growth and resistance against nematodes. (**A**) Seedling growth of three overexpressed lines (T3-5/7/10) versus controls (WT, GFP) grown on MS medium for 14 days. (**B**,**C**) Quantitative analysis of root length (**B**) and fresh root weight (**C**) (*n* = 45). (**D**) Shoot growth after growing in soil for 28 days. (**E**) Gall numbers per plant at 35 dpi (*n* = 45). (**F**,**G**). Infection phenotypes on WT root (**F**) vs. transgenic line root (**G**) at 35 dpi. Scale bar = 1 cm. Error bars represent SE. Different superscripts indicate significant difference (Games–Howell’s test, *p* < 0.05).

To investigate the direct impacts of the nematode SQS-PSY domain-containing protein on the host plant, the full-length CDS of *Minc31999* was amplified and inserted into the Super1300-HA vector to generate an expression construct (Super1300-HA-*Minc31999*). This construct was then transformed into *A. thaliana* via ATMT. Positive transformants were confirmed by PCR, RT-qPCR, and Western blot analysis (Supplementary Figure S6). The growth and development of these transgenic lines were then compared to control plants (WT and GFP transgenic plants). Seedlings grown on MS medium for 14 days showed that overexpression of *Minc31999* significantly impaired root development. The root length of overexpressed lines (mean of 5.1 to 5.3 cm) was shorter than that of the controls (mean of 6.9 cm for WT and 7.0 cm for GFP transgenic line) (Figure 6A,B), and their root weight of overexpressed lines (mean of 75.3 to 80.6 mg) was reduced compared to controls (mean of 110.7 mg for WT and 112.5 mg for GFP transgenic line) (Figure 6C). Furthermore, when grown in soil for 28 days, the shoots of the *Minc31999*-overexpressed lines were visibly less vigorous than control plants (Figure 6D). We next assessed whether this nematode protein affects plant resistance. Following inoculation with *M. incognita* J2s, the *Minc31999*-overexpressed lines developed significantly more galls (mean 40 galls per plant) at 35 dpi than control plants (mean 28 per plant) (Figure 6E). Consequently, root damage was also substantially more severe in the overexpression lines (Figure 6F,G). Collectively, these results demonstrate that the nematode SQS-PSY domain-containing protein encoded by *Minc31999* not only suppresses plant growth and development but also enhances host susceptibility to root-knot nematode infection.

### 2.7. Differentially Expressed Genes (DEGs) Between Minc31999 Overexpressed Lines and WT A. thaliana

**Figure 7:**
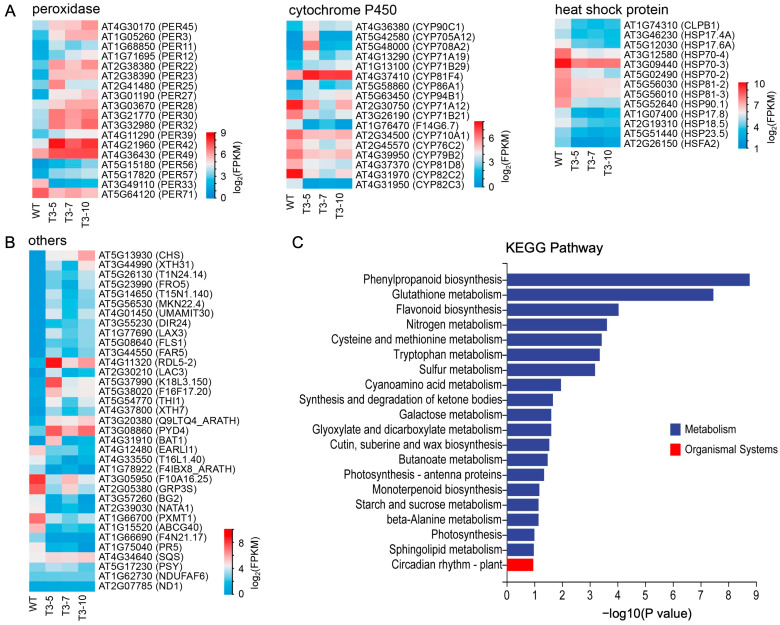
Transcriptomic profiling of *Minc31999* overexpressed *A. thaliana* lines. (**A**) Heatmaps of differentially expressed genes (DEGs) in key functional categories, namely peroxidases (16↑, 2↓), cytochrome P450 proteins (8↑, 9↓), and heat shock proteins (13↓). (**B**) Expression patterns of additional significant DEGs and SQS-PSY-related genes. (**C**) Top 20 enriched KEGG pathways from DEG analysis. Data derived from RNA-seq of three independent overexpression lines (T3-5/7/10) and WT plants with three biological replicates each.

To explore the molecular mechanisms by which the nematode SQS-PSY domain-containing protein affects plant growth and development, we performed RNA-seq to obtain transcriptomes from the roots of three *Minc31999* overexpressed lines (T3-5, T3-7, and T3-10) and the WT *A. thaliana* plants, each with three biological replicates. Comparative transcriptomics identified 1306 differentially expressed genes (DEGs) between the overexpressed lines and the WT, comprising 923 up-regulated and 383 down-regulated genes (Supplementary Figure S7). Notably, 837 up-regulated and 364 down-regulated DEGs were consistent across all three overexpressed transgenic lines (Supplementary Tables S2 and S3). Gene ontology analysis revealed pronounced up-regulated genes, including those encoding peroxidases (sixteen genes, Figure 7A), cytochrome P450 family proteins (eight genes, Figure 7A), glycosyl hydrolases (eight genes), bifunctional inhibitor/lipid-transfer protein/seed storage 2S albumin superfamily proteins (eight genes), TRAF-like family proteins (six genes), S-adenosyl-L-methionine-dependent methyltransferases superfamily proteins (six genes), polyketide cyclase/dehydrase and lipid transport superfamily proteins (five genes), MLP-like proteins (five genes), serine carboxypeptidases (five genes), cysteine proteinases (five genes), xyloglucan endotransglucosylases/hydrolases (five genes), 2-oxoglutarate (2OG), Fe(II)-dependent oxygenase superfamily proteins (five genes), and so on (Supplementary Table S2). Conversely, several key gene families were significantly down-regulated, including encoded heat shock proteins (HSPs, twelve genes) and heat shock transcription factor (Figure 7A), cytochrome P450 family proteins (nine genes, Figure 7A), UDP-glucosyl transferases (five genes), NAD(P)-binding Rossmann-fold superfamily proteins (five genes), and others (Supplementary Table S3). Nevertheless, the expression of the three endogenous *Arabidopsis* SQS-PSY domain-containing genes (*AT4G34640*/SQS, *AT5G17230*/PSY, *AT1G62730*/NDUFAF6) remained unchanged across the different lines (Figure 7B). As NDUFA6 is crucial for mitochondrial complex I assembly via regulating ND1 subunit biogenesis, we also examined the expression of the ND1-coding gene (*AT2G07785*), which was similarly unchanged (Figure 7B). This indicates that the nematode SQS-PSY domain-containing protein encoded by *Minc31999* does not function as a plant NDUFA6 homolog in this pathway.

KEGG analysis revealed that the identified DEGs are predominantly enriched in metabolic pathways. These include fundamental processes such as the biosynthesis of secondary metabolites, carbon metabolism, amino acid biosynthesis, nucleotide metabolism, and cofactor biosynthesis. Specifically, the key pathways involved were phenylpropanoid biosynthesis (ko00940), glutathione metabolism (ko00480), flavonoid biosynthesis (ko00941), nitrogen metabolism (ko00910), cysteine and methionine metabolism (ko00270), tryptophan metabolism (ko00380), sulfur metabolism (ko00920), and cyanoamino acid metabolism (ko00460) (Figure 7C). Additionally, some DEGs were implicated in signal transduction pathways, including plant hormone signal transduction (ko04075), the plant MAPK signaling pathway (ko04016), and the NOD-like receptor signaling pathway (ko04621), as well as in plant–pathogen interaction (ko04626).

Among the top up- and down-regulated DEGs (Figure 7B), the most significantly up-regulated was *AT5G13930*, which encodes chalcone synthase (CHS), a key enzyme in the flavonoid biosynthesis pathway (ko00941). Several other genes in this pathway were also markedly upregulated, including those encoding chalcone isomerases (CHIL and TT5; *AT5G05270*, *AT3G55120*), flavonol synthases (FLS3 and FLS5; *AT5G63590*, *AT5G63600*), and flavanone 3-hydroxylase (F3H; *AT3G51240*). Flavonoids are a major class of plant secondary metabolites that serves multiple functions, including acting as pigments and antioxidants. The coordinated up-regulation of these genes suggests a strong activation of flavonoid production. Notably, 15 of the 16 up-regulated peroxidase genes are involved in phenylpropanoid biosynthesis (ko00940), a pathway linked to flavonoid and lignin production. Peroxidases contribute to oxidation–reduction reactions, oxidative stress responses, and plant defense. Other up-regulated genes in this pathway contribute to lignin monomer synthesis and cell wall lignification, including those encoding UDPG/coniferyl alcohol glucosyltransferase (UGT72E2; *AT5G66690*), aldehyde dehydrogenase (ALDH2C4; *AT3G24503*), and laccases (LAC3 and LAC7; *AT2G30210*, *AT3G09220*). Lignin is a crucial structural component of the cell wall, vital for plant growth and environmental adaptability. Two TGA transcription factors (TGA3 and TGA1; *AT1G22070*, *AT5G65210*), which have dual roles in plant development and defense, were markedly upregulated. Three genes (*AT5G26130*, *AT4G33720*, *AT4G07820*) encoding CAP (cysteine-rich secretory proteins, antigen 5, and pathogenesis-related 1 protein) superfamily proteins (CAP32, CAP37, CAP39) were also highly upregulated, though their functions remain unknown. The antioxidative defense system was bolstered by the upregulation of superoxide dismutase (SOD1; *AT2G28190*) and catalase (CAT2; *AT4G35090*), which protect plants against reactive oxygen species (ROS) generated by biotic and abiotic stress. In addition, most DEGs involved in signaling pathways were clearly up-regulated. These genes mainly participate in plant hormone signal transduction (ko04075), such as those encoding CPK (*AT3G19100*), AUX1 (*AT5G01240*, *AT1G77690*), TGA (*AT1G22070*), IAA (*AT5G43700*, *AT2G22670*, *AT4G29080*), ARF (*AT5G60450*), CH3 (*AT1G28130*), SNRK2 (*AT2G23030*), and NDK (*AT4G11010*). Among the downregulated DEGs, the most significantly repressed gene was the pathogenesis-related gene *PR5* (*AT1G75040*). The heat shock transcription factor A2 (HSFA2; *AT2G26150*), a master regulator of stress responses, was also significantly down-regulated. Consequently, the expressions of 12 heat shock protein (HSP) genes were significantly reduced, including members of the HSP100 (*AT1G74310*), HSP90 (*AT5G52640*, *AT5G56010*, *AT5G56030*), HSP70 (*AT5G02490*, *AT3G12580*, *AT3G09440*), and small HSP (*AT2G19310*, *AT1G07400*, *AT5G51440*, *AT5G12030*, *AT3G46230*) families. As molecular chaperones, HSPs are pivotal for stress tolerance, and Hsp90 also participates in the NOD-like receptor signaling pathway (ko04621) and plant–pathogen interaction (ko04626). In conclusion, the overexpression of *Minc31999* in *A. thaliana* significantly alters gene expression, primarily affecting metabolic processes, especially the biosynthesis of secondary metabolites like flavonoids and lignin. These changes likely impact plant growth, resistance, and the regulation of signal transduction systems and stress responses.

We validated the RNA-seq expression patterns of 16 key DEGs (8 up-regulated and 8 down-regulated) using RT-qPCR on the same *A. thaliana* lines, confirming the transcriptomic data (Supplementary Figure S8).

## 3. Discussion

### 3.1. Evolutionary Conservation of the SQS-PSY Domain-Containing Protein in Nematoda

Currently, SQS-PSY domain-containing proteins, including squalene synthase, phytoene synthase, and NDUFAF6, are known to be present in plants, animals, fungi, and bacteria. However, their existence has not been reported in nematodes. This gap was likely due to the low sequence similarity between nematode SQS-PSY proteins and other known proteins in this family, making a homolog difficult to establish. In this study, we first identified the protein-coding gene (*Minc31999*) in the *M. incognita* genome via an antiSMASH analysis of secondary metabolite biosynthetic gene clusters. It was initially predicted to encode a phytoene synthase, a key enzyme for carotenoid biosynthesis, which is uncommon in animals. Pfam annotation confirmed its membership in the squalene synthase/phytoene synthase family (PF00494). We then retrieved known SQS-PSY domain-containing proteins from the three model species (*H. sapiens*, *D. melanogaster*, and *A. thaliana*) from NCBI for structural and sequence analysis. The nematode protein shared less than 25% sequence identity with any known homolog. Based on sequence similarity, active site motif conservation, and spatial structures, we propose that the nematode protein is more closely related to NDUFAF6 than to SQS or PSY. This was supported experimentally, as the protein lacks the catalytic activity of both SQS and PSY. Furthermore, we identified homologous sequences in other nematode genomes. Phylogenetic analysis revealed that these nematode proteins form a distinct monophyletic group, which is clearly separate from the clade containing NDUFAF6 from humans, fruit flies, and *Arabidopsis* (Figure 3, Supplementary Figure S3). These results indicate that the SQS-PSY domain-containing proteins are conserved within the phylum Nematoda but are evolutionarily distinct from known NDUFAF6 proteins.

### 3.2. The SQS-PSY Domain-Containing Protein Encoded by Minc31999 May Act as an Effector to Be Involved in Nematode–Plant Interaction

NDUFAF6 was first reported in humans, and NDUFAF6 mutants are associated with complex I enzymatic deficiency, resulting in a range of human diseases [12,13,14]. In this study, RT-qPCR analysis revealed that *Minc31999* expression was highest during the early parasitic stage of the *M. incognita* life cycle. Down-regulating the *Minc31999* expression through HIGS impaired nematode development and reduced pathogenicity (Figure 5). Conversely, overexpressing *Minc31999* in *A. thaliana* altered plant growth and compromised resistance against *M. incognita* (Figure 6). These results indicate that the NDUFAF6-like protein encoded by *Minc31999* has acquired a novel function in the root-knot nematode, potentially acting as an effector in plant–nematode interaction. A key question is how this nematode protein is secreted into the host plant, as it lacks a signal peptide and is not expressed in the esophageal glands, indicating it is not a conventional esophageal gland effector. In situ hybridization localized the expression of *Minc31999* throughout the nematode’s body, including the amphidial and phasmidial regions, epidermis, and hypodermis (Figure 4C). Nematodes are known to secrete effectors through multiple routes, including the esophageal gland, amphids, phasmids, and hypodermis [17]. Like esophageal gland secretions, cuticular secretions from the hypodermis and epidermis can play a crucial role in suppressing plant defenses. Therefore, we propose that the SQS-PSY domain-containing protein encoded by *Minc31999* may be secreted into the host via cuticular secretion. This mechanism would be similar to that of MIFs in *M. incognita*, which were reported to be secreted in planta from the nematode hypodermis to the plant–parasite interface [18]. However, this hypothesis requires further experimental validation.

### 3.3. Putative Mechanisms of Minc31999 Involved in Nematode–Plant Interaction

To understand the mechanisms by which the nematode protein acts on its plant host, the *Minc31999* gene was introduced into the *A. thaliana* genome using transgenic technology, leading to nematode SQS-PSY protein expression. Subsequently, RNA-seq and comparison analysis identified DEGs between WT and transgenic lines. KEGG functional annotation revealed that the majority of DEGs were primarily involved in metabolic pathways, especially the biosynthesis of secondary metabolites such as flavonoids and the phenylpropanoids lignin, sulfur, and glutathione (Figure 7C). The production of these secondary metabolites, which serve various functions as structural molecules, signaling molecules, phytotoxins, and antioxidants, influences plant growth, stress tolerance, and pathogen resistance. Among the upregulated genes, chalcone synthase (CHS), which catalyzes the first committed step in flavonoid biosynthesis, was highly enhanced. CHS can also act on dihydro-4-coumaroyl-CoA to form phloretin, a key secondary metabolite found in the roots. Reported as a phytotoxic allelochemical, phloretin inhibits growth in *Arabidopsis* by disrupting auxin metabolism, and it can significantly hinder seedling shoot development by inducing chloroplast damage and programmed cell death [19]. Moreover, genes encoding peroxidases, UGTs, and laccases, all of which are involved in lignin metabolism, were highly expressed. Lignin, a major structural component of vascular plant cell walls, is integral to plant growth, development, and disease resistance. Enhancement of gene expression in lignin biosynthesis and subsequent lignin accumulation may improve plant resistance to pathogens but can also reduce the plant growth rate due to the trade-offs between growth and defense [20]. Conversely, genes involved in pathogen response were significantly downregulated in the transgenic lines. The PR5 protein, a thaumatin-like protein, plays a critical role in plant defense against pathogens and antibiotics [21]. For instance, overexpression of *AtPR-5* in soybean roots reduced the number of cysts formed by the soybean cyst nematode (*Heterodera glycines*) to 38% of the control [22]. We found that the expression of the PR5-coding gene (*AT1G75040*) was significantly down-regulated. The expression levels of the heat shock transcription factor HsfA2 and 12 heat shock proteins were also markedly reduced. HSFs functions as transcriptional activators controlling HSP expression, and *HsfA2* transcript levels are known to increase significantly under various stress conditions or following H_2_O_2_ treatment [23]. Low levels of HSP expression may decrease plant resistance against nematodes.

Reactive oxygen species (ROS), including hydrogen peroxide (H_2_O_2_), superoxide anions (O_2_^−^), and hydroxyl radicals (OH•), are generated in the plants during cellular metabolism and accumulate under biotic and abiotic stress. Plants counteract this through defense systems that limit ROS formation and facilitate its removal. We found that genes encoding superoxide dismutase (SOD1) and catalase (CAT2) were highly expressed in the *Minc31999* ectopic expression lines, indicating oxidative stress and activation of the antioxidant defense system to scavenge ROS. To confirm that ROS accumulation was reduced by the high expression of SOD1 and CAT2, we measured superoxide anion (O_2_^−^) and hydrogen peroxide (H_2_O_2_) contents in different *Arabidopsis* lines. As expected, both were obviously reduced in the overexpressed lines (Supplementary Figure S9).

**Figure 8:**
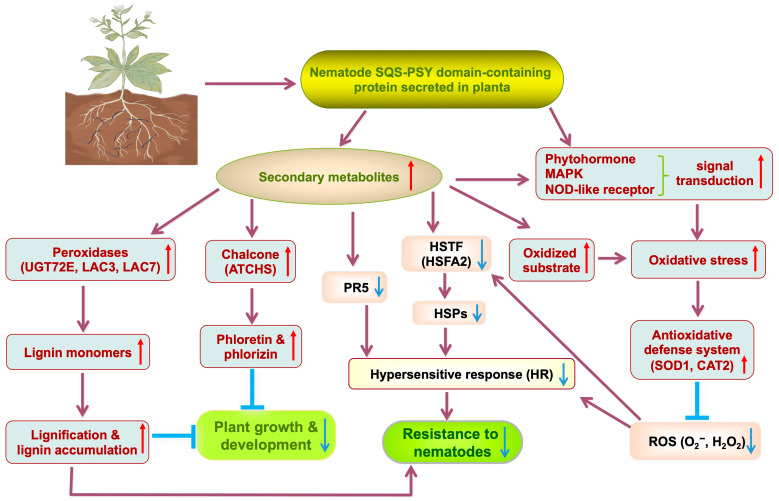
Proposed mechanism of *Minc31999* in nematode–plant interaction. The SQS-PSY domain-containing protein encoding by *Minc31999*, secreted by *M. incognita*, may function as an effector that modulates host interactions by altering host secondary metabolism, suppressing plant defense responses and inhibiting plant growth and development to facilitate the nematode parasitism.

Based on current transcriptomic data, we propose a model for the role of the nematode SQS-PSY domain-containing protein in plant–nematode interaction (Figure 8). We suggest that during *M. incognita* infection onto roots, the nematode protein enters the host’s cells by cuticular secretions. Functioning as an effector, it interacts with host proteins, altering the host’s metabolism and biosynthesis of secondary metabolites. The accumulation of phytotoxic allelochemicals like phloretin inhibits plant growth and may increase lignin deposition. Simultaneously, some secondary metabolites may act as signaling molecules, activating signal transduction pathways that trigger oxidative stress and subsequently activate the antioxidative defense system. The high expression of superoxide dismutase (SOD1) and catalase (CAT2) scavenges ROS (O_2_^−^ and H_2_O_2_), and reduced ROS levels may impair the plant’s hypersensitive response (HR), diminishing resistance. Furthermore, the significant suppression of genes encoding HSPs, HSFA2, and PR5 may also compromise the HR and reduce resistance against nematode infection.

The proposed model is based on our current transcriptomic profiling of *Minc31999*-overexpressed *Arabidopsis* in this study. However, as our interpretation relied on selecting a subset of biologically relevant genes from over a thousand DEGs, it is inherently limited and likely to overlook critical genes and pathways. To address these limitations and provide a more mechanistic understanding, future efforts should aim to identify direct host protein interactors for Minc31999. Techniques such as co-immunoprecipitation (Co-IP) followed by LC-MS/MS assays, in conjunction with yeast two-hybrid (Y2H) assays, will be essential for future studies.

## 4. Materials and Methods

### 4.1. Nematode and Plant Materials

The RKN *M. incognita*, which was originally isolated from tomato plant (*Solanum lycopersicum*) and maintained long-term in our lab (IVF, CAAS, Beijing, China) following morphology and molecular identification, was multiplied on the water spinach (*Ipomoea aquatica*). Egg masses were picked from infected roots, placed in distilled water, and incubated at 28 °C. Hatched second-stage juveniles (J2s) were collected at 24 h intervals. Seeds of *A. thaliana* (ecotype Columbia, Col-0) were surface-sterilized and cultured on Murashige and Skoog (MS) solid medium (Coolaber, Beijing, China) for 14 days. Germinated seedlings were then transplanted into pots containing a 3:1 mixture of soil and vermiculite and cultured at 22 °C under a 16 h light/8 h dark photoperiod.

### 4.2. Bioinformatics Analysis

The gene structure of *Minc31999* was determined using Gene Structure Display Server v2.0 (https://gsds.gao-lab.org/, accessed on 18 November 2024). Using the Pfam database v27.0 (https://pfam.xfam.org/, accessed on 25 February 2023), we identified all genes containing the SQS-PSY domain in several representative plant and animal species by performing a search with HMMER software v3.1b2 (http://hmmer.org/, accessed on 25 February 2023). The RoseTTAFold Server v1.1.0 (https://robetta.bakerlab.org, accessed on 28 February 2025) was used to predict the protein spatial structure [24]. Protein structures were compared using TM-align v2022-04-15 (https://zhanggroup.org/TM-align/, accessed on 3 March 2025), and the results were visualized with Jmol v16.3.33 (http://jmol.sourceforge.net/, accessed on 3 March 2025). Published nematode genomes were downloaded from NCBI (www.ncbi.nlm.nih.gov, accessed on 12 June 2021) and WormBase ParaSite WS291 (https://parasite.wormbase.org/, accessed on 12 June 2021) databases. We used BLASTp v2.2.26 (E-value < 1 × 10^−5^) [25] to identify homologous sequences of *Minc31999* in nematodes by aligning its amino acid sequence against the protein sequences of each downloaded genome. Multiple sequence alignments were performed using MUSCLE v3.8.31 [26]. The phylogenetic trees were constructed with IQ-TREE v1.6.12 using the maximum likelihood algorithm [27], and node support was assessed with 1000 bootstrap replicates. The resulting trees were visualized using iTOL v7.2 [28]. The motif active sites of the domain sequences were predicted using MEME v5.5.8 (https://meme-suite.org/meme/tools/meme, accessed on 2 October 2024). RNA-seq transcriptome data for five different developmental stages of *M. incognita* were retrieved from the NCBI. The CDS of *Minc31999* was then aligned to this transcriptome data using BLASTn (E-value < 1 × 10^−5^).

### 4.3. RNA Extraction and Gene Cloning

The freshly hatched J2 juveniles were collected and concentrated to a final volume of approximately 50 μL. Total RNA was then extracted from *M. incognita* using TRIzol reagent (Ambion, Carlsbad, CA, USA). The RNA concentration and purity were measured with a spectrophotometer, and its integrity was confirmed by agarose gel electrophoresis. First-strand cDNA was synthesized from the extracted RNA using FastKing gDNA Dispelling RT Super Mix (Tiangen Biotech, Beijing, China), according to the manufacturer’s instructions.

Gene-specific primers were designed based on the predicted coding sequence (CDS) of *M. incognita* (Supplementary Table S4). The synthesized cDNA was used as a template for PCR amplification. The 50 μL PCR reactions were performed using Phanta Max Master Mix (Vazyme Biotech, Nanjing, China) with the following cycling parameters: an initial denaturation at 95 °C for 3 min; 35 cycles of 95 °C for 15 s, 60 °C for 15 s, and 72 °C for 30 s; and a final elongation at 72 °C for 5 min. The PCR products were purified using the Fast Pure Gel DNA Extraction Kit (Vazyme Biotech, Nanjing, China) according to the manufacturer’s protocol. The purified fragments were ligated into a vector and transformed using *pEASY*-Blunt Simple Cloning Kit (TransGen Biotech, Beijing, China). Positive single colonies were selected and sent for sequencing to TSINGKE Biological Technology (Beijing, China).

### 4.4. Heterologous Expression and Purification of Protein

The protein encoded by *Minc31999* was heterologously expressed in *Escherichia coli*, based on methods from previous studies [29,30]. The full-length CDS of *Minc31999* was ligated into the pMAL-c5X vector linearized by *EcoR*I and *Hind*III. The resulting recombinant plasmid was transformed into the *E. coli* BL21 (DE3) expression strain. The primers used are listed in Supplementary Table S4. Five monoclonal colonies were selected and cultured in LB medium at 37 °C with shaking at 200 rpm until the OD_600_ reached 0.6–0.8. Protein expression was then induced by adding 0.5 mM isopropyl β-d-1-thiogalactopyranoside (IPTG). After continuous cultivation for 3 h at 37 °C, the cells were harvested by centrifugation and lysed. Protein expression was verified by SDS-PAGE and Coomassie blue staining. To optimize expression, a gradient of IPTG concentrations was tested at induction temperatures of 37 °C and 16 °C. Bacterial cultures were centrifuged, and the pellets were sonicated. The supernatant and pellet fractions were prepared and analyzed by SDS-PAGE. Due to the low yield of soluble protein in the supernatant, the culture volume was scaled up to 1 L and induced with IPTG at 16 °C for 15 h. The cells were harvested, lysed by sonication, and the supernatant was purified by affinity chromatography with MBP tag. The equilibration and wash buffer were PBS (pH 7.4), and the elution buffer was PBS with 10 mM maltose (pH 7.4). The purified protein was verified by SDS-PAGE and Western blot analysis using an anti-MBP tag monoclonal antibody. Additionally, the CDS of *AT4G34640*, which encodes the SQS protein in *A. thaliana*, was also heterologously expressed in *E. coli* using the pET-30a vector following established methods [31]. The primers are listed in Supplementary Table S4. The soluble protein was purified by His-tag affinity column chromatography and used as a positive control in an enzyme activity assay.

### 4.5. Enzymatic Assay In Vitro

Enzymatic activity was measured in a 100 μL reaction volume containing 50 mM HEPES buffer (pH 7.5), 25 μM substrate, and 20 μg purified protein. Activity was assayed using four substrates, (*E,E*)-FPP (Sigma), (*Z,Z*)-FPP (Echelon), (*Z,E*)-FPP (Echelon), and GGPP (Sigma). Reactions were incubated at 30 °C for 2 h; Mg^2+^ was included for FPP substrates and Mn^2+^ for GGPP [8]. After incubation, reaction products were extracted and analyzed by gas chromatography–mass spectrometry (GC-MS) and liquid chromatography–mass spectrometry (LC-MS) [32]. For GC-MS analysis, 200 μL of hexane was added to the reaction mixture, which was then vortexed for 1 min and centrifuged at 4000 rpm for 5 min. The hexane layer was transferred to a glass vial for analysis [33]. For LC-MS analysis, 100 μL of methanol was added, and the mixture was vortexed and transferred directly to a vial. All enzyme assays were performed with three biological replicates, each with at least three technical replicates for mass spectrometry detections.

GC-MS was performed on a Shimadzu GC/MS-QP 2010 Ultra system. The oven temperature was programmed as follows: an initial temperature of 60 °C, increased to 380 °C at a rate of 5 °C/min, and held at 380 °C for 5 min. The injection volume was 1 μL, and the helium carrier gas flow rate was maintained at 1 mL/min [34]. LC-MS was performed using an Agilent 1260 HPLC system coupled to an Agilent MS-Q-TOF 6520 mass spectrometer. The mobile phase consisted of (A) 5 mM NH_4_HCO_3_ in water and (B) acetonitrile, with a flow rate of 0.2 mL/min and a column temperature of 30 °C [35]. The gradient program was as follows: 0–20 min, 95% to 0% A; 20–25 min, 0% A; 25–25.1 min, 0% to 95% A; 25.1–31 min, 95% A for column equilibration. The injection volume was 10 μL. Detection was performed using negative electrospray ionization (ESI) with fragmentor and capillary voltages set to 125 V and 3500 V, respectively. Nitrogen was used as the nebulizing and drying gas at a temperature of 300 °C and a flow rate of 11 L/min; the nebulizer pressure was 45 psi. Full-scan spectra were acquired over a range of *m*/*z* 80–2000 at a rate of 1.03 spectra per second [36].

### 4.6. In Situ Hybridization

In situ hybridization (ISH) was performed on freshly hatched *M. incognita* J2 juveniles using digoxigenin labeling [37]. Sense and antisense probes were synthesized based on a specific sequence of the *Minc31999* gene (5′-CAGGTTGACGAGGCAACAGAGCATCTCTTCTAGCTAACGAATTTAAAGCATCATTCC-3′). Hybridization signals were detected using a mouse anti-digoxin antibody conjugated to horseradish peroxidase, with diaminobenzidine (DAB) as the substrate [38]. This reaction produced a brown precipitate in the target area. Nuclei were counterstained with hematoxylin, which stained them a bluish-purple color. Stained nematodes were observed under an Olympus BX51 microscope (Japan).

### 4.7. Observation of M. incognita Development in Plant

A large number of *M. incognita* J2s were inoculated into the roots of wild-type (WT) *A. thaliana*. Root samples were collected at 1, 3, 5, 7, 14, 21, and 28 dpi. Roots were stained with acid fuchsin solution (3.5g/L) [39], and nematode development within the roots was observed using an Olympus IX53 microscope (Japan).

### 4.8. RT-qPCR

Total RNA was extracted from *A. thaliana* root samples infected with *M. incognita* using TRIzol reagent. cDNA was synthesized from the extracted RNA using HiScript III RT SuperMix for qPCR (+gDNA wiper) (Vazyme Biotech, Nanjing, China). Reverse transcription quantitative real-time PCR (RT-qPCR) was then performed using Taq Pro Universal SYBR qPCR Master Mix (Vazyme Biotech, Nanjing, China). The *GAPDH* gene of *M. incognita* was used as an internal reference. The relative expression level of *Minc31999* at different time points after *M. incognita* infection in *A. thaliana* was calculated using the 2^−ΔΔCT^ method [40]. The experiment included at least three biological replicates, each with three technical replicates. The primers used for RT-qPCR are listed in Supplementary Table S4.

### 4.9. Construction of RNA Interference Vector and Host-Induced Gene Silencing (HIGS)

To construct the RNA interference vector, a 291 bp target fragment of *Minc31999* was amplified by PCR from a cDNA template. This fragment was inserted into the pSAT5 vector [41] in both sense and antisense orientations, flanking an intermediate spacer sequence. This design enables the formation of a small hairpin RNA (shRNA) molecule during transcription. The entire shRNA expression cassette, containing the upstream and downstream target fragments and the spacer, was then excised from pSAT5 using *Xba*I and *Kpn*I and ligated into the linearized plant expression vector pSuper to create the final construct for *A. thaliana* expression. A control vector, pSuper-*GFP*-RNAi, was constructed in parallel using the GFP encoding sequence. The primers used are listed in Supplementary Table S4.

Recombinant plasmids, verified by sequencing, were transformed into *Agrobacterium tumefaciens* GV3101 competent cells (Zoman Biotechnology, Beijing, China). WT *A. thaliana* plants were transformed via the floral dip method using this *Agrobacterium* strain [42]. Transgenic plants from the T1 and T2 generations were selected on MS plates containing hygromycin, and integration of the transgene was confirmed by genomic PCR. For the host-induced gene silencing (HIGS) assay [43], 300 *M. incognita* J2 juveniles were inoculated onto each positive T3 generation transgenic plants. WT and GFP-RNAi lines served as negative controls. To evaluate the silencing effect, *Minc31999* expression levels in nematode feeding on different lines were analyzed by RT-qPCR at 3 dpi at 3, 7, and 21 dpi. The HIGS phenotype was further investigated by inoculating 200 J2 juveniles onto each of three independent transgenic lines and the control lines. At 28 dpi, nematodes within the roots were stained with acid fuchsin solution, and the width of the female nematodes was measured. At 42 dpi, the number of galls and egg masses was counted, and the fresh root weight was measured. All experiments were performed with three biological replicates, each containing at least 15 plants.

### 4.10. Overexpression of Minc31999 in A. thaliana and Phenotype Observation

The *Minc31999* overexpression vector was constructed using the Super1300-HA vector [44]. The full-length CDS was ligated into the Super1300-HA vector linearized with *Sal*I and *Kpn*I. The resulting recombinant plasmid was introduced into *A. thaliana* using the same method described previously. A GFP-Super1300-HA vector was used as a negative control. Positive transformed lines were selected on MS plates containing hygromycin. Integration of the *Minc31999* transgene was confirmed by genomic PCR in the T1 generation plants, and its expression level was verified by RT-qPCR in T2 generation plants. The primers used are listed in Supplementary Table S4. The expression of the Minc31999 protein in T3 generation lines was confirmed by Western blot analysis using an anti-HA monoclonal antibody. To assess phenotypic effects, *A. thaliana* seedlings were grown on MS plates for 14 days, after which the root lengths of WT, GFP control, and overexpressed lines were measured. Seedlings were then transplanted into nutrient soil. After 35 days of growth, the fresh root weight of the different lines was measured. For nematode infection assays, 200 *M. incognita* J2 juveniles were inoculated onto the roots of these plants. and the number of galls was counted at 35 dpi. All experiments were performed with three biological replicates, each containing at least 15 plants per line.

### 4.11. RNA-Seq and Transcriptome Analysis

Root samples from the three overexpressed lines (T3-5, T3-7, and T3-10) and WT *A. thaliana* were collected for RNA sequencing. Sequencing was performed on the Illumina platform by Bluescape Hebei Biotech Co., Ltd. (Baoding, China), with three replicates per line. The *A. thaliana* TAIR10 genome sequences and annotation were used as the reference for analysis (https://www.arabidopsis.org, accessed on 8 March 2024). Gene expression analysis followed a previously described protocol [45]. Briefly, reads were aligned to the reference genome using Hisat2 v2.2.1 [46]. The resulting SAM files were converted to BAM files using SMAtools v1.16.1 [47]. Stringtie v2.2.1 [48] was then used to analyze the alignment and calculate gene expression values in FPKM. Differentially expressed genes (DEGs) between the WT and each transgenic line (T3-5, T3-7, T3-10) were identified based on the FPKM values. The R functions fisher.test, p.adjust, and log_2_ were used to calculate *p*-values, false discovery rate (FDR) values (using the “BH” method [49]), and log_2_(FoldChange) values, respectively. DEGs were defined as genes with an adjusted *p*-value (FDR) ≤ 0.01 and an absolute log_2_(FoldChange) ≥ 1. KEGG annotation was performed for all expressed genes. The metabolic pathways involving the DEGs were identified by querying their respective KO numbers (https://www.genome.jp/kegg/ko.html, accessed on 6 February 2025). Heatmaps of gene expression were generated using TBtools v2.330 [50]. To validate the transcriptome data, the expression levels of eight up-regulated DEGs and eight down-regulated DEGs were confirmed by RT-qPCR.

### 4.12. Reactive Oxygen Species (ROS) Burst Detection

To quantify reactive oxygen species (ROS), the levels of superoxide anion (O_2_^−^) and hydrogen peroxide (H_2_O_2_) were measured in the leaves of three overexpressed lines (T3-5, T3-7, and T3-10) and control lines (WT and GFP). A total of 100 mg of leaf tissue was homogenized in 1 mL of the appropriate treatment solution on ice. The homogenate was centrifugated at 4 °C, and the resulting supernatant was collected for analysis. The superoxide anion content was determined using a commercial assay kit (Boxbio, Beijing, China). The supernatant was put through a reaction according to the manufacturer’s instructions, and the absorbance was measured at 530 nm. The concentration was calculated based on a standard curve prepared for the assay. Similarly, the hydrogen peroxide content was measured using its respective assay kit (Boxbio, Beijing, China). Following the kit protocol, the absorbance of the reaction mixture was read at 415 nm, and the H_2_O_2_ concentration was determined using a corresponding standard curve.

### 4.13. Statistical Analysis

All statistical analyses were conducted with GraphPad Prism (version 8.3.0). Datasets were first tested for Gaussian (normal) distribution. Subsequently, significant differences were analyzed by a one-way ANOVA. Data visualization was also performed using GraphPad Prism.

## 5. Conclusions

In conclusion, we identified a gene (*Minc31999*) encoding an SQS-PSY domain-containing protein in the nematode *M. incognita*. Structural and enzymatic analyses revealed that this protein is enzymatically inactive as a squalene or phytoene synthase and is a putative NDUFAF6 homolog. Phylogenetic analysis showed that this SQS-PSY domain-containing protein is evolutionarily conserved within the Nematoda phylum. Expression profiling revealed that *Minc31999* is highly expressed in the early parasitic J2 stage and is localized to diverse nematode tissues, including the amphidial and phasmidial regions, epidermis, and hypodermis. Functional studies confirmed that this protein is not only critical for nematode development and parasitism but also impairs the host’s growth and resistance against nematodes. RNA-seq analysis showed that Minc31999 expression in transgenic plants altered the expression of numerous host genes involved in diverse biochemical pathways. Collectively, our findings indicate that the nematode SQS-PSY domain-containing protein is likely a novel effector protein that promotes parasitism through its dual role in nematode development and modulating host cellular processes.

## Figures and Tables

**Table 1:** BLAST hits of *Minc31999* in *M. incognita* transcriptomes of different life stages.

Life Stage	SRA Accession	Sequence ID	Score (Bits)	E-Value
Egg	SRX2923455	SRA:SRR5689337.31485.1	1701	<1 × 10^−300^
J2	SRX2923454	SRA:SRR5689335.29893.1	1620	<1 × 10^−300^
J3	SRX2923453	SRA:SRR5689336.25751.1	939	<1 × 10^−300^
J4	SRX2923452	SRA:SRR5689338.12770.1	1602	<1 × 10^−300^
Female	SRX2923451	SRA:SRR5689339.21014.1	1638	<1 × 10^−300^

## Data Availability

The raw data of the transcriptome in this study has been submitted to the NCBI SRA database under project number PRJNA1226758.

## Supplementary Materials

The supporting information can be downloaded at: https://www.mdpi.com/article/10.3390/ijms26189113/s1.

## Abbreviations

The following abbreviations are used in this manuscript:

RKN	Root-Knot Nematode
SQS	Squalene Synthase
PSY	Phytoene Synthase
MS medium	Murashige and Skoog Medium
GC-MS	Gas Chromatography–Mass Spectrometry
LC-MS	Liquid Chromatography–Mass Spectrometry
HIGS	Host-Induced Gene Silencing
DEGs	Differentially Expressed Genes
ROS	Reactive Oxygen Species
ATMT	Agrobacterium Tumefaciens-Mediated Transformation
CHS	Chalcone Synthase
HSP	Heat Shock Protein
