# Supplementary material for: Discovery of an SQS-PSY Domain-Containing Protein in Meloidogyne incognita Reveals Its Function in Parasitism
Source: Int J Mol Sci. 2025 Sep 18;26(18):9113. doi: 10.3390/ijms26189113 (PMC12470011; doi:10.3390/ijms26189113)
Supplement: Supplementary file 1 [file ijms-26-09113-s001.zip › ijms-3842080-supplementary/Suppl Data.pdf]

>Minc31999-gene

ATGTTTATTTCTTCTATAAATAAAACAATAAAAACAACACAGCCAACAACAA
CATTAAAATCAACTCCACAGCAATATCAAGAAGCTTTAAATTATTGTGTTAA
AATTGTAAAAGAAAGGGATTATGAAAATTATTTGGCTGCTTTATTAATGCCT
TCAAATGTTAGGGCAAAAATATTTACGGTTTTCTTTTTTAAATTAATTTTAAA
AAATTATTTTCTTTTAATTTTAAAGATTTTGGCGTTAAATGCAGAAATATCTA
CTCTGAGGCACAAAATAAAGAGGCACTCTGGCGTTGCAAATATCAATCAAT
TAAAGTTTTGGAATGATGCTTTAAATTCGTTAGCTAGAAGAGATGCTCTGTT
GCCTCGGTTTTTTTTTATTTATTTTAATTTTGGGGATTTTTTTATCGGATTTTC
TTGTAAATTTCGACTTTTTTACTAGCATTCTGAACTATTTTCAGATTTTAGTA
AAAGATATTAGCCTTAAATTGACAAATTATTTAATACCGTTGTTGTCCGAATG
GTATCGTTCTGGAGTTGCCCTCTAAGGAGGGTGCAGGTTCGCTTCCTCGCT
TGGATTATTTGCCTTTGTTTTTTGCTTTTTTGTTCTAAAATTCTTGTAAAATCT
TTTTAAAAATTTTCCCAGGCCTTAAAAATTTTTTCTTGAAAATTTTCGACTC
ACCCGCTACCCTTTAATTTTTTAAAAATTTTTACAACTCTAACAAAAAAATT
AATTTTCTTTTCAGTCAACCTGTAATTCTCGCCCTCCAAGCATTTTACAATTT
TCCCCAGCAATCATCTACAGAATTTAATTTATTTTTAAATTTAATAAAATCAA
GACAACAAACTTTGGGTGATCGTCCTTTTGAAAATTTAAATTTTTTAGAAA
ATTATTGTAAATTACTTTATGGCTCTTTAATTTTGCTTCAAATGAATTGTTTAA
ATAGACAAGAAGTTCAACTTGTAGATGAGTCAAAATTGGAAAAAGTTGTAA
ATTCAATAGCTTGTTCTATTGGTATATTAACTTTGTTGAGGTTTTCTTTTTTAA
ATTTTTTAAATTAAAAAAATTTTTTTTCTTAAGAGCAACCACTTTACTGTTAA
AACATGATGGAATTGTGCTTCTCCCACAAGATGTGACAGACCTGCATGGGT
TCAATATTGATTCTGCTGCTCGTAGTGGGCCTAATGCAATGAAAGATGTTGC
TAAAGATTTGTGCAAGGTTTTGAAATTTTTTAAAATAATTTTTGTTGGTTATT
CCTGCTTGGTTTTGAGCAAGTTTTGGGGACTGGGTCTAGAGGTTCGCTTTA
ATATGGGAGTTGCCTTGAACTCAGGCTTTTTTCGAGCTTCCTGGGTTTTTTC
TGAATAAATGCCCGGATTCTTAACCCCTTATTTAGACGGGGCAAGACCATTG
GTAAACTTTGGGATGTTTCTGAACGTTTCCCGAGTGATGTTCTTAAGTCCCT
GTCTTCCATTATCCGTATTTTCATCTAGGAGGAATTTCTTTGATAACCAGCAG

CCTTAAACAGTTCGTGTTTACCTATGCTTTCCCCCTCAATATTTATTTTTTCTA
GATAGCTTCTACTCATCTCAATTCTGCGCGATCCTTAACATGTTCAATTCATC
CTTCTTTAAGGCCAGCCTTGTTAACTGTAGGATTTCGTTCTGATTGGATTTTA
AAGGTTTTAGAAAAATCAGATTTTAATTTGTTGGATGTTCGACTACATAGAA
GACAACACCCTTTTGCTGCTTTAATACTTTGGTGGAGATATAGAAGAAGAAT
ATTTTAA

>Minc31999-CDS

ATGTTTATTTCTTCTATAAATAAAACAATAAAAACAACACAGCCAACAACAA
CATTAAAATCAACTCCACAGCAATATCAAGAAGCTTTAAATTATTGTGTTAA
AATTGTAAAAGAAAGGGATTATGAAAATTATTTGGCTGCTTTATTAATGCCT
TCAAATGTTAGGGCAAAAATATTTACGATTTTGGCGTTAAATGCAGAAATAT
CTACTCTGAGGCACAAAATAAAGAGGCACTCTGGCGTTGCAAATATCAATC
AATTAAAGTTTTGGAATGATGCTTTAAATTCGTTAGCTAGAAGAGATGCTCT
GTTGCCTCGTCAACCTGTAATTCTCGCCCTCCAAGCATTTTACAATTTTCCC
CAGCAATCATCTACAGAATTTAATTTATTTTTAAATTTAATAAAATCAAGACA
ACAAACTTTGGGTGATCGTCCTTTTGAAAATTTAAATTTTTTAGAAAATTAT
TGTAAATTACTTTATGGCTCTTTAATTTTGCTTCAAATGAATTGTTTAAATAG
ACAAGAAGTTCAACTTGTAGATGAGTCAAAATTGGAAAAAGTTGTAAATTC
AATAGCTTGTTCTATTGGTATATTAACTTTGTTGAGAGCAACCACTTTACTGT
TAAAACATGATGGAATTGTGCTTCTCCCACAAGATGTGACAGACCTGCATG
GGTTCAATATTGATTCTGCTGCTCGTAGTGGGCCTAATGCAATGAAAGATGT
TGCTAAAGATTTGTGCAAGATAGCTTCTACTCATCTCAATTCTGCGCGATCC
TTAACATGTTCAATTCATCCTTCTTTAAGGCCAGCCTTGTTAACTGTAGGATT
TCGTTCTGATTGGATTTTAAAGGTTTTAGAAAAATCAGATTTTAATTTGTTG
GATGTTCGACTACATAGAAGACAACACCCTTTTGCTGCTTTAATACTTTGGT
GGAGATATAGAAGAAGAATATTTTAA

>Minc31999-aa

MFISSINKTIKTTQPTTTLKSTPQQYQEALNYCVKIVKERDYENYLAALLMPS
NVRAKIFTILALNAEISTLRHKIKRHSGVANINQLKFWNDALNSLARRDALLPR
QPVILALQAFYNFPQQSSTEFNLFLNLIKSRQQTLGDRPFENLNFLENYCKLLY

GSLILLQMNCLNRQEVQLVDESKLEKVVNSIACSIGILTLLRATTLLLKHDGIV
LLPQDVTDLHGFNIDSAARSGPNAMKDVAKDLCKIASTHLNSARSLTCSIHPS
LRPALLTVGFRSDWILKVLEKSDFNLLDVRLHRRQHPFAALILWWRYRRRIF
