# Supplementary figures and images for: Discovery of an SQS-PSY Domain-Containing Protein in Meloidogyne incognita Reveals Its Function in Parasitism
Source: Int J Mol Sci. 2025 Sep 18;26(18):9113. doi: 10.3390/ijms26189113 (PMC12470011; doi:10.3390/ijms26189113)

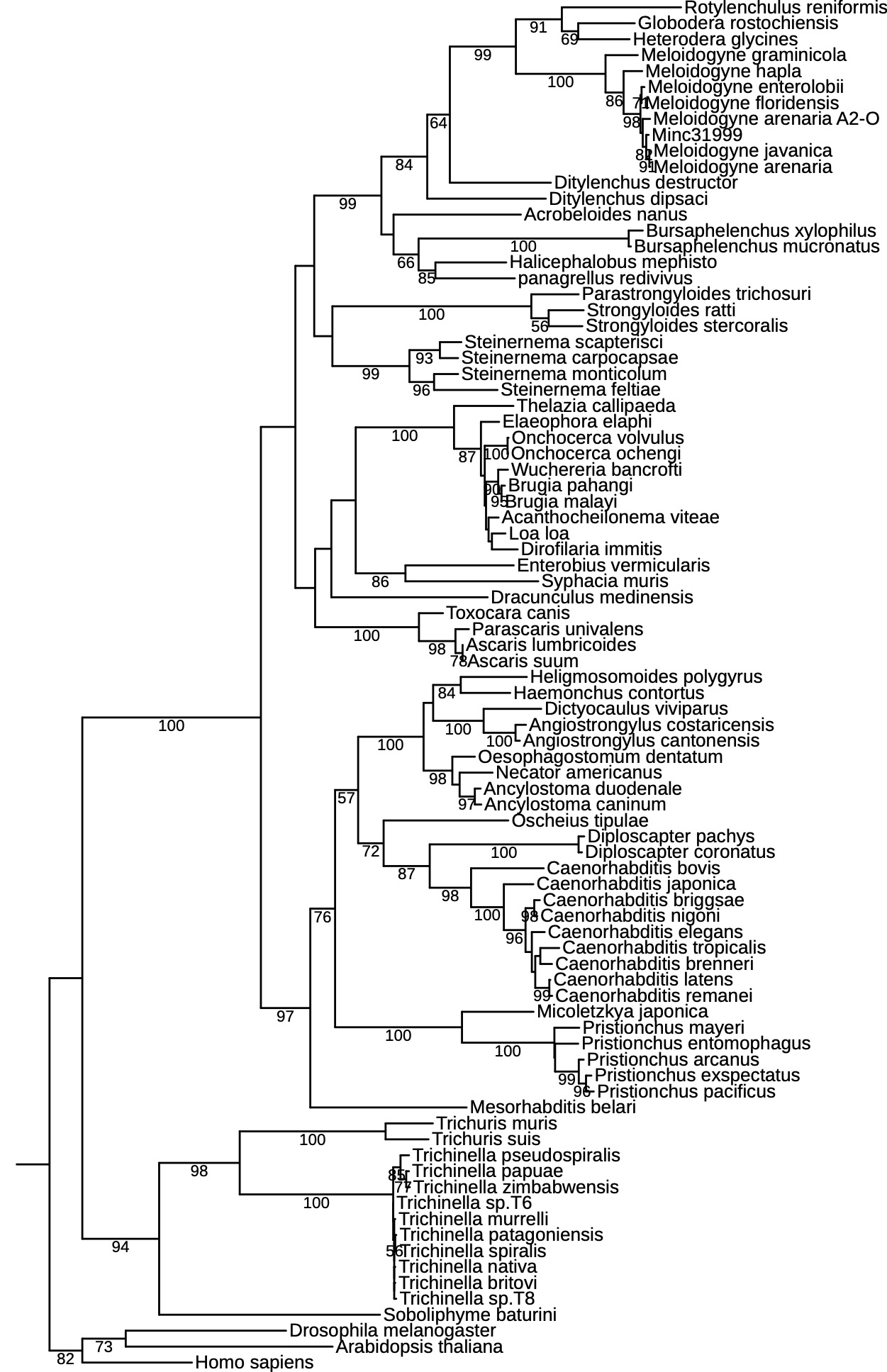

Supplement: Supplementary file 1 [file ijms-26-09113-s001.zip › ijms-3842080-supplementary/suppl Figs/Fig S3.tif]

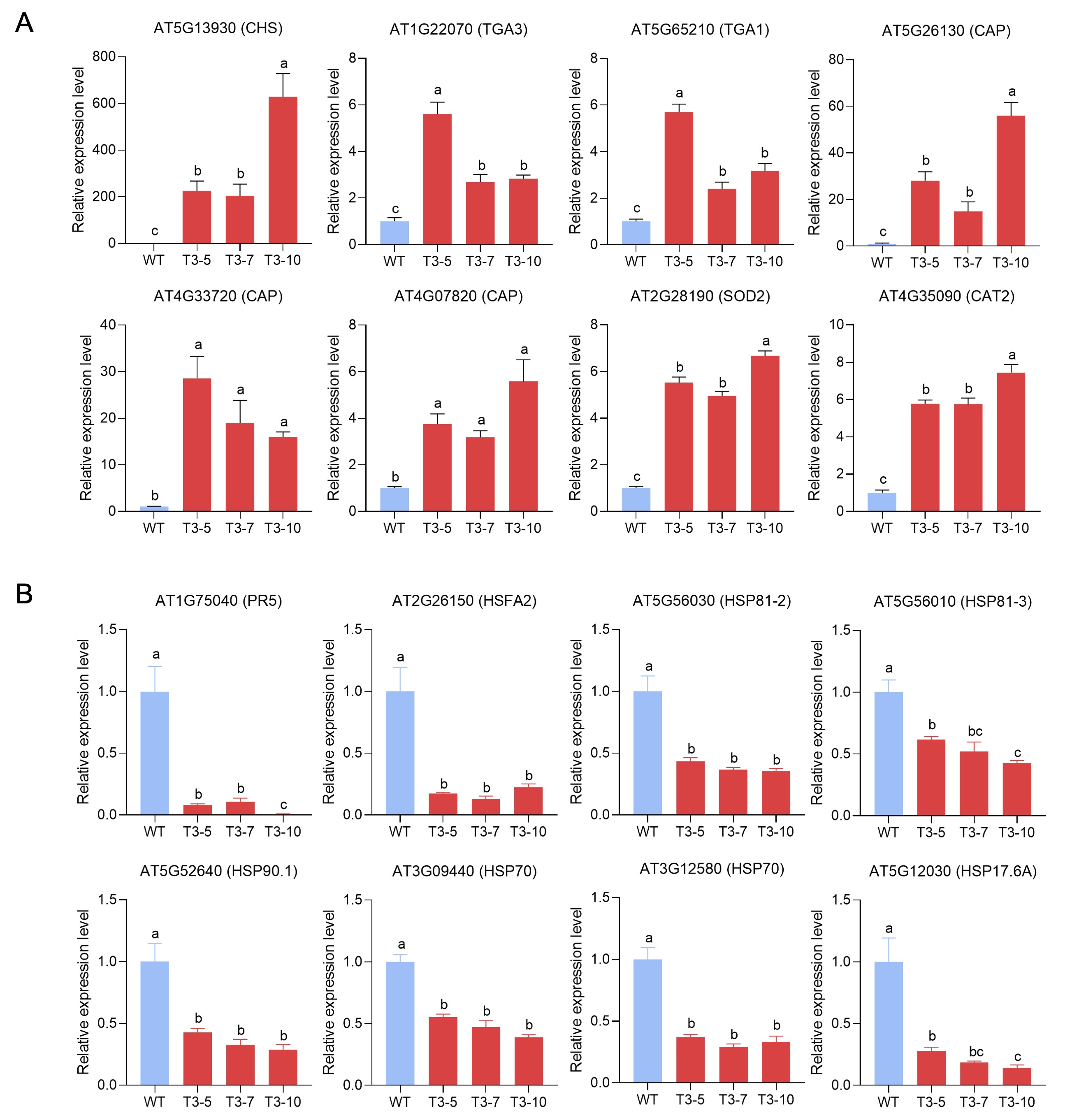

Supplement: Supplementary file 1 [file ijms-26-09113-s001.zip › ijms-3842080-supplementary/suppl Figs/Fig S8.tif]

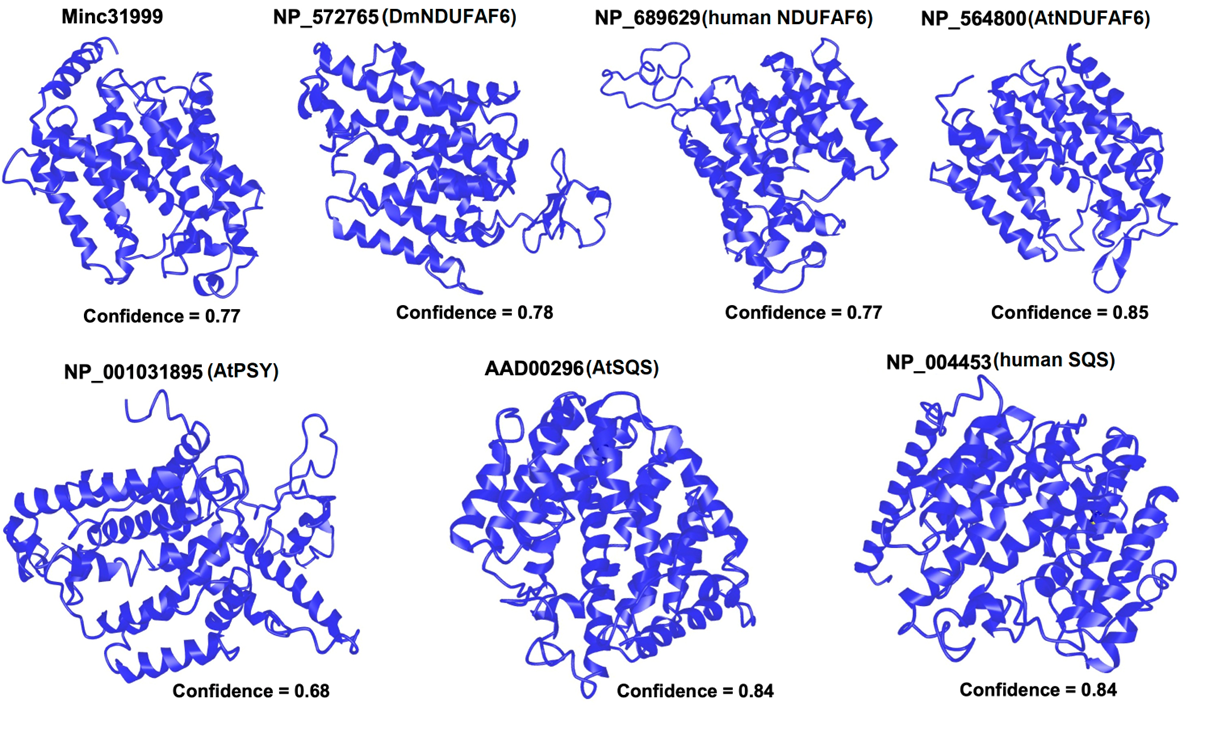

Supplement: Supplementary file 1 [file ijms-26-09113-s001.zip › ijms-3842080-supplementary/suppl Figs/FigS1.tif]

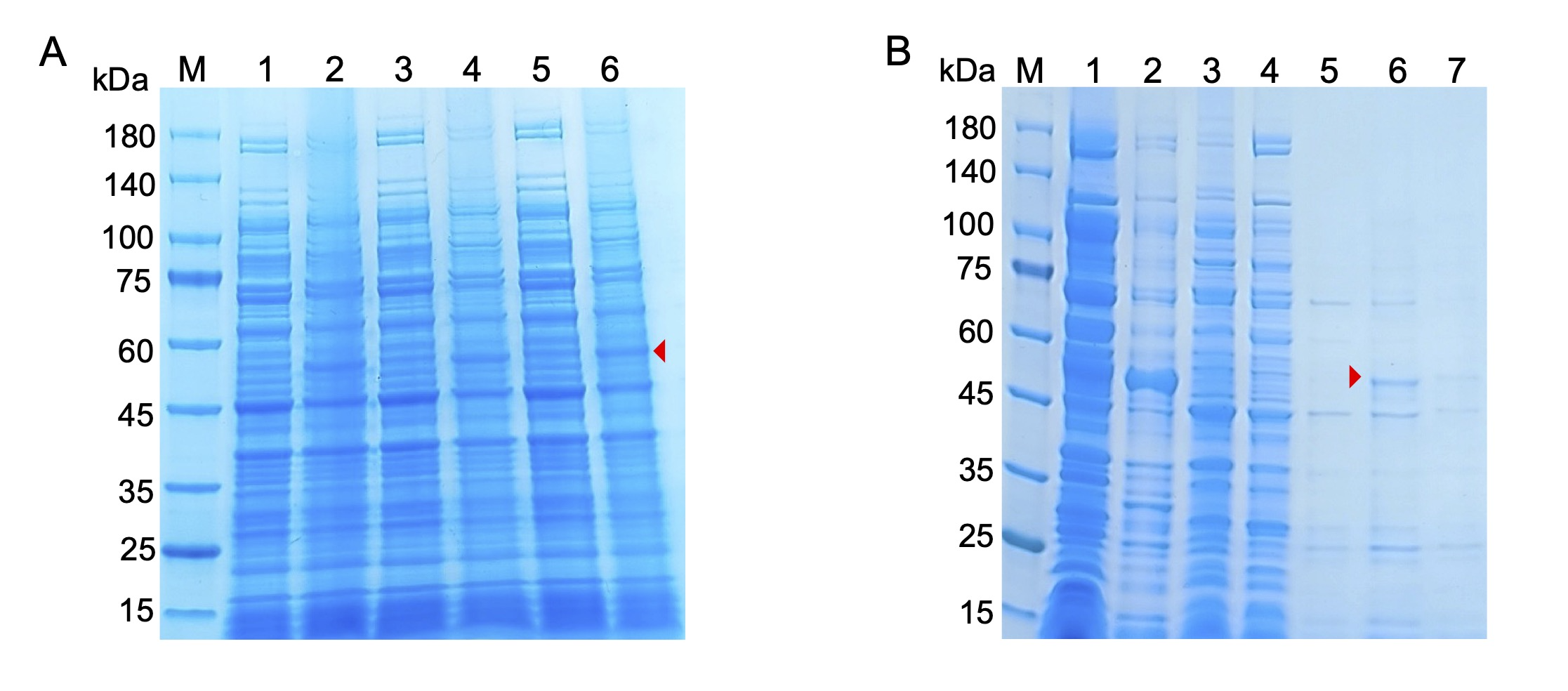

Supplement: Supplementary file 1 [file ijms-26-09113-s001.zip › ijms-3842080-supplementary/suppl Figs/FigS2.tif]

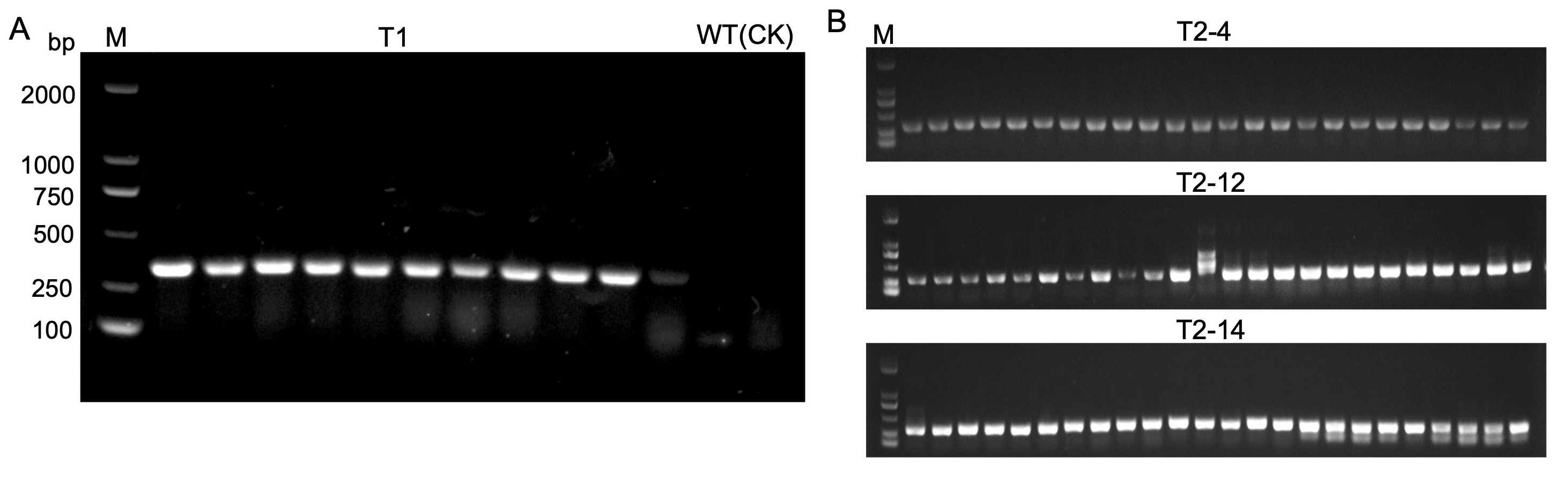

Supplement: Supplementary file 1 [file ijms-26-09113-s001.zip › ijms-3842080-supplementary/suppl Figs/FigS4.tif]

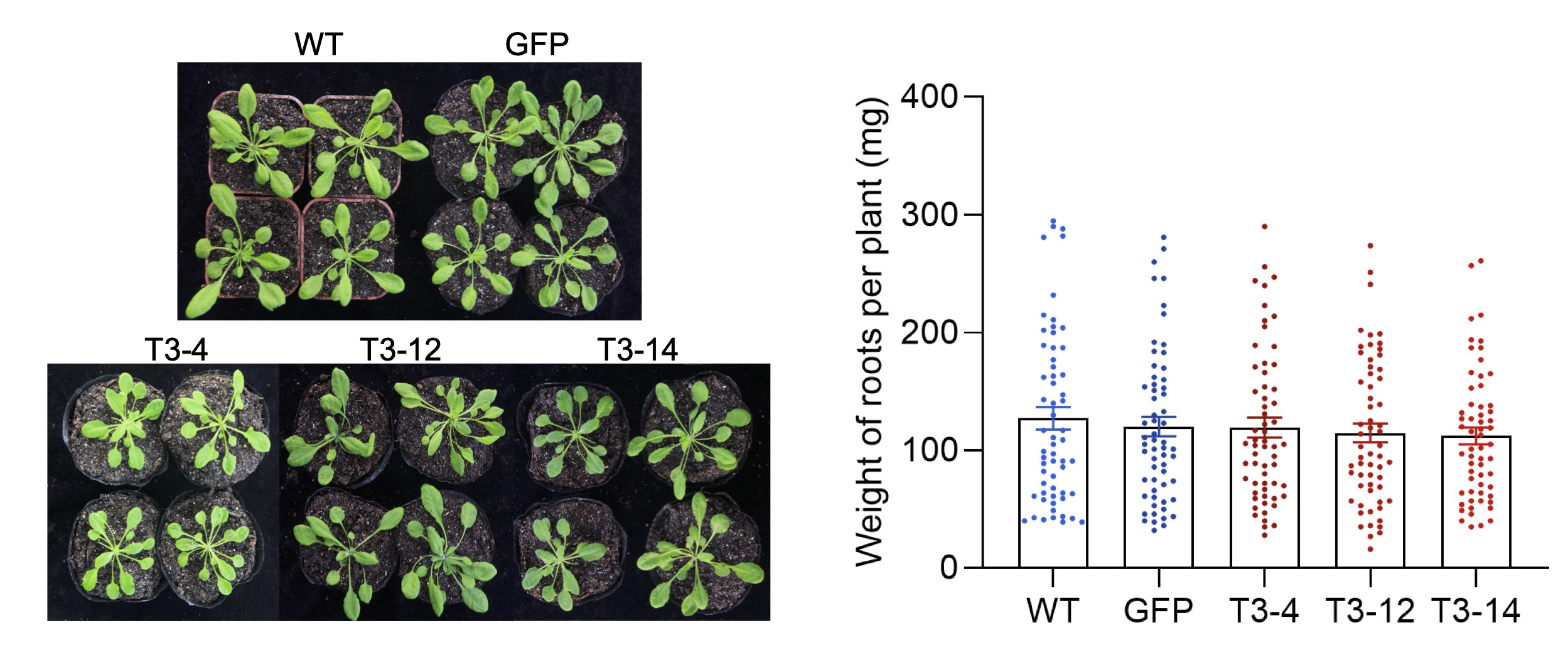

Supplement: Supplementary file 1 [file ijms-26-09113-s001.zip › ijms-3842080-supplementary/suppl Figs/FigS5.tif]

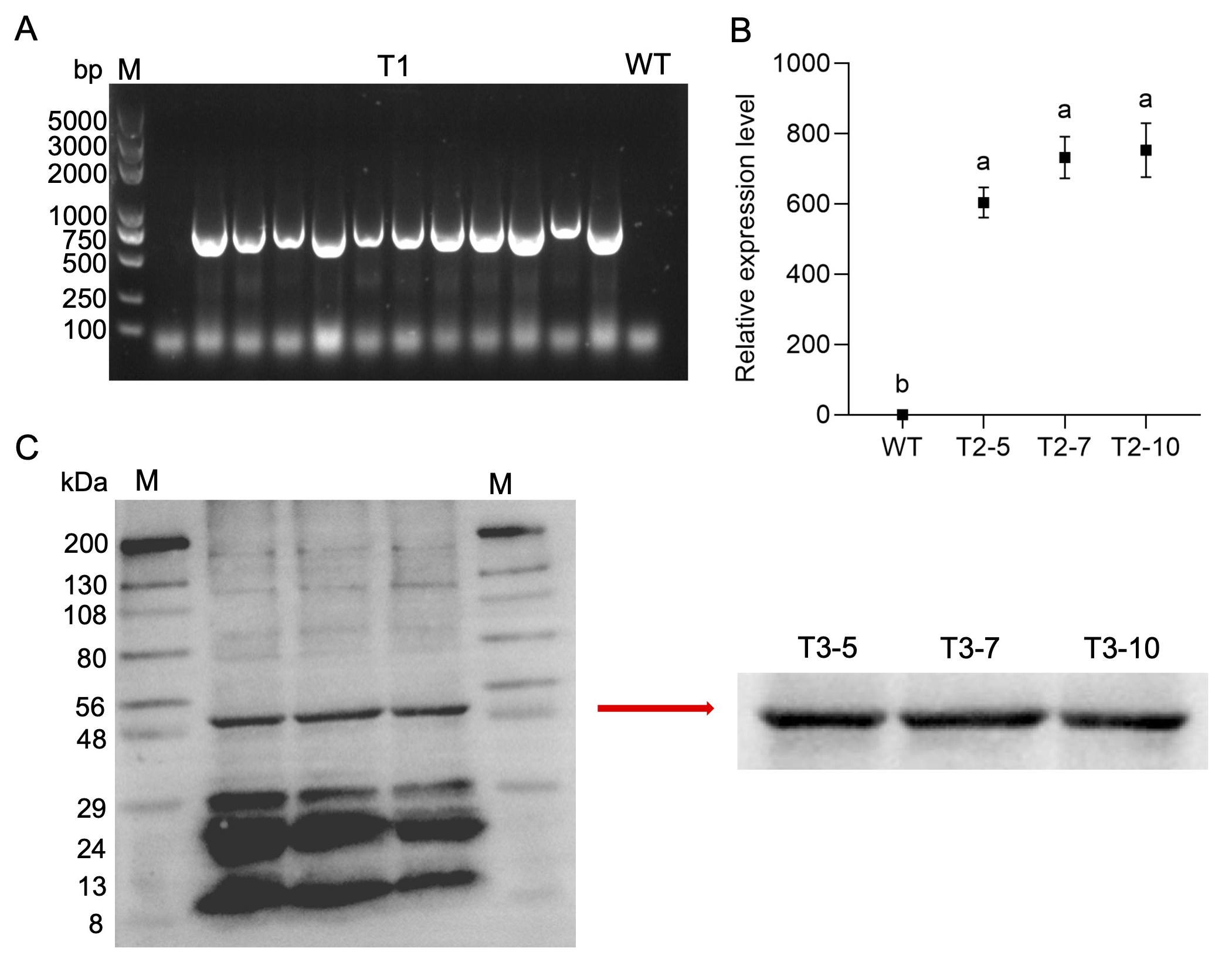

Supplement: Supplementary file 1 [file ijms-26-09113-s001.zip › ijms-3842080-supplementary/suppl Figs/FigS6.tif]

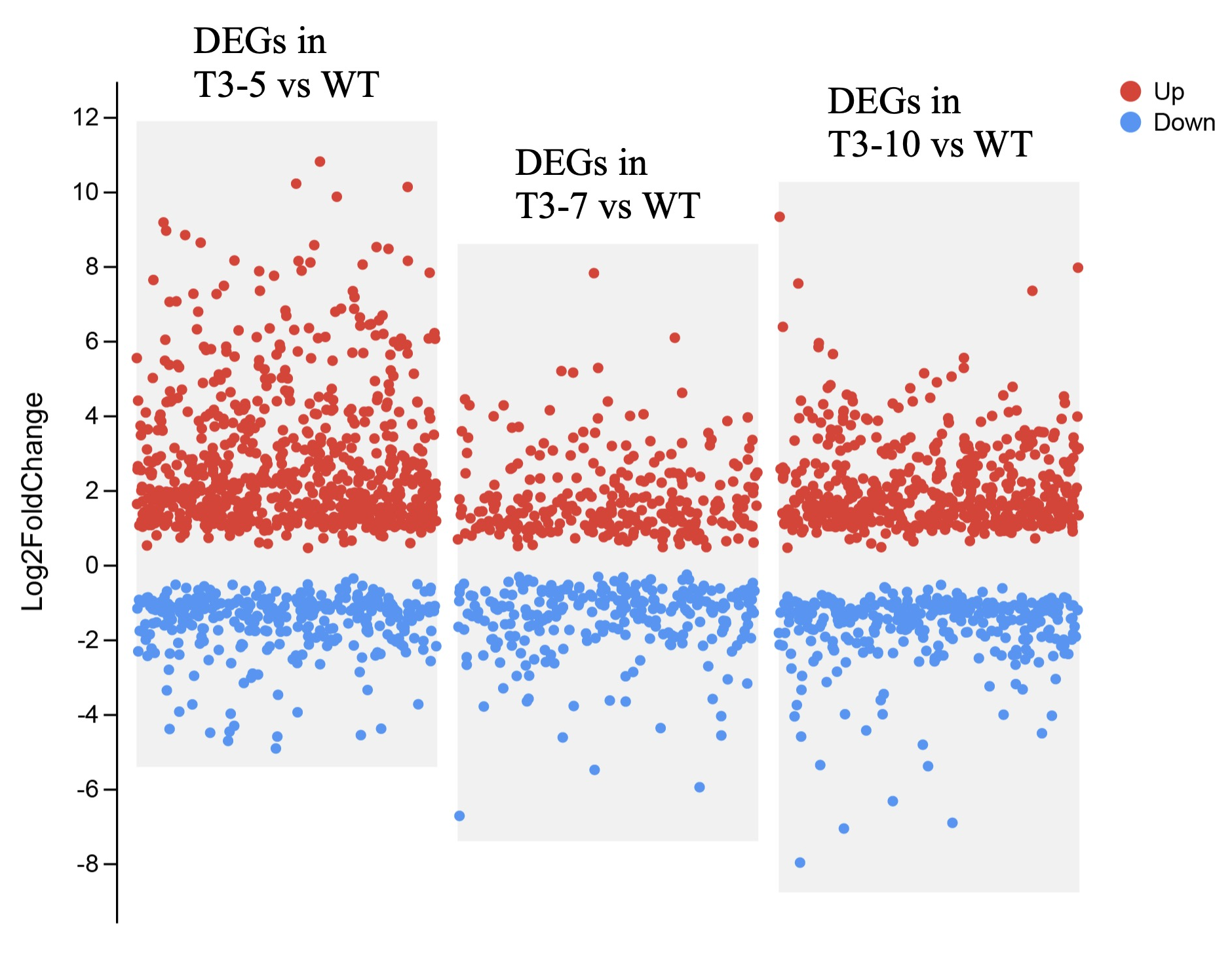

Supplement: Supplementary file 1 [file ijms-26-09113-s001.zip › ijms-3842080-supplementary/suppl Figs/FigS7.tif]

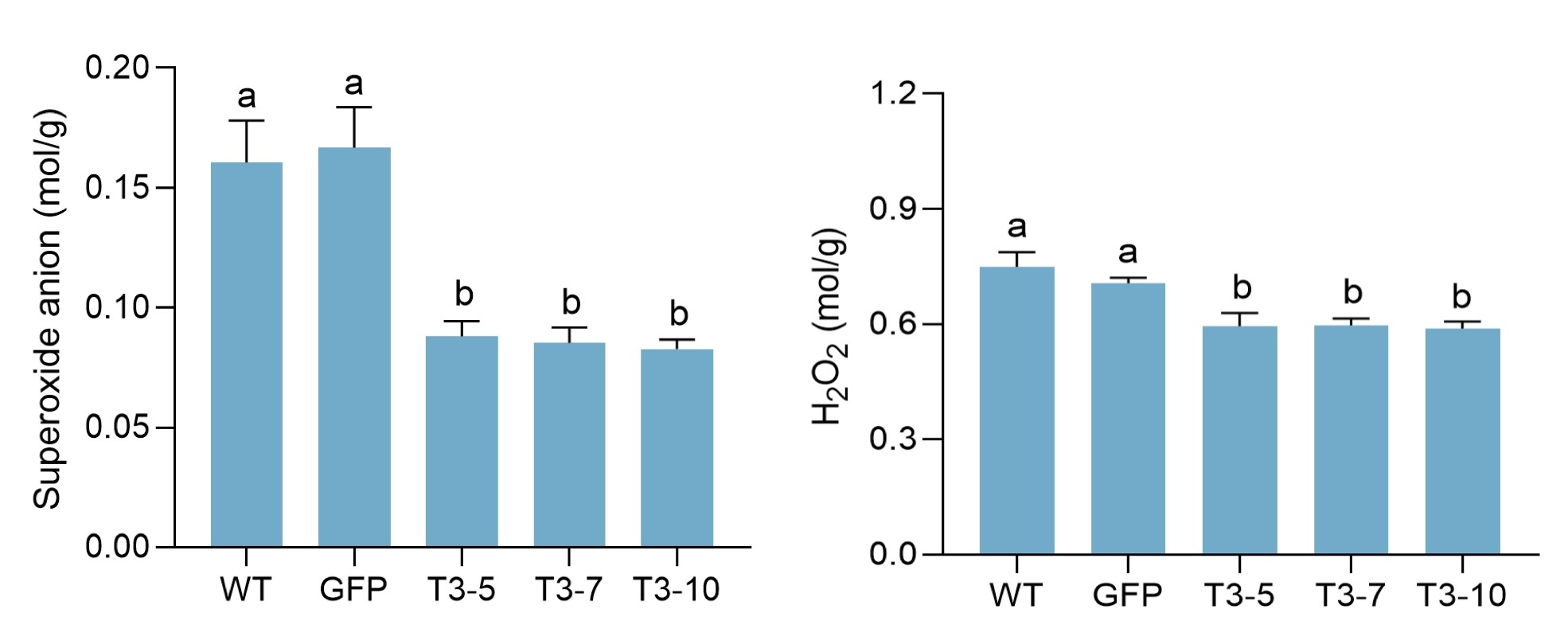

Supplement: Supplementary file 1 [file ijms-26-09113-s001.zip › ijms-3842080-supplementary/suppl Figs/FigS9.tif]
